# The Adverse Effects of Environmental Noise Exposure on Oxidative Stress and Cardiovascular Risk

**DOI:** 10.1089/ars.2017.7118

**Published:** 2018-03-20

**Authors:** Thomas Münzel, Mette Sørensen, Frank Schmidt, Erwin Schmidt, Sebastian Steven, Swenja Kröller-Schön, Andreas Daiber

**Affiliations:** ^1^ The Center for Cardiology, Cardiology 1, Johannes Gutenberg University Medical Center, Mainz, Germany.; ^2^ Danish Cancer Society Research Center, Copenhagen, Denmark.; ^3^ Institute for Molecular Genetics, Johannes Gutenberg University, Mainz, Germany.

**Keywords:** environmental risk factors, traffic noise exposure, aircraft noise exposure, stress hormones, endothelial dysfunction, oxidative stress

## Abstract

Epidemiological studies have provided evidence that traffic noise exposure is linked to cardiovascular diseases such as arterial hypertension, myocardial infarction, and stroke. Noise is a nonspecific stressor that activates the autonomous nervous system and endocrine signaling. According to the noise reaction model introduced by Babisch and colleagues, chronic low levels of noise can cause so-called nonauditory effects, such as disturbances of activity, sleep, and communication, which can trigger a number of emotional responses, including annoyance and subsequent stress. Chronic stress in turn is associated with cardiovascular risk factors, comprising increased blood pressure and dyslipidemia, increased blood viscosity and blood glucose, and activation of blood clotting factors, in animal models and humans. Persistent chronic noise exposure increases the risk of cardiometabolic diseases, including arterial hypertension, coronary artery disease, diabetes mellitus type 2, and stroke. Recently, we demonstrated that aircraft noise exposure during nighttime can induce endothelial dysfunction in healthy subjects and is even more pronounced in coronary artery disease patients. Importantly, impaired endothelial function was ameliorated by acute oral treatment with the antioxidant vitamin C, suggesting that excessive production of reactive oxygen species contributes to this phenomenon. More recently, we introduced a novel animal model of aircraft noise exposure characterizing the underlying molecular mechanisms leading to noise-dependent adverse oxidative stress-related effects on the vasculature. With the present review, we want to provide an overview of epidemiological, translational clinical, and preclinical noise research addressing the nonauditory, adverse effects of noise exposure with focus on oxidative stress. *Antioxid. Redox Signal.* 28, 873–908.

## Table of Contents

I. Introduction

A. Noise and global burden of disease

B. Historical view on noise research: the concept of nonauditory effects of noise

C. Impact of environmental noise on healthcare systems

II. Epidemiology: Traffic Noise and Cardiometabolic Disease

A. Cardiovascular disease

B. Metabolic disease

C. Cancer

D. Effects of noise exposure on sleep: short sleep, endothelial dysfunction, and oxidative stress

E. Traffic noise exposure, annoyance, noise sensitivity, and mental disease

F. Effects of noise and air pollution coexposure

G. Health effects of noise exposure in children

H. Traffic noise mitigation strategies

III. Effects of Noise on Vascular Function and Oxidative Stress

A. Prognostic meaning of endothelial dysfunction and vascular oxidative stress

B. Noise and translational studies in humans

C. Noise and translational studies in animals

1. Direct pathway activation *via* ≥100 dB(A) noise exposure on the inner ear (hearing loss)

2. Indirect, nonauditory vascular effects of ≤100 dB(A) noise exposure

3. Indirect, nonauditory pathway activation with noise exposure ≤85 dB(A)

4. Effects of aircraft noise on vascular gene regulation as established by next-generation sequencing

5. Summary and conclusions of aircraft noise exposure in mice

IV. Adverse Effects of Simultaneous Noise and Air Pollution Exposure

A. Adverse effects of noise and air pollution exposure share similar pathophysiological pathways

B. Gaps in current knowledge concerning noise and air pollution

V. Other Environmental Risk Factors, Oxidative Stress, and Cardiovascular Disease

A. Air pollution (PM/carbon black)

B. Other environmental stressors

VI. Summary and Future Perspectives

## I. Introduction

During the last decades, there was a shift of the global burden of disease from communicable (*e.g.*, of perinatal, nutritional nature) to noncommunicable causes (*e.g.*, atherosclerosis) (143). Whereas most research was directed toward classical risk factors such as diabetes, smoking, or arterial hypertension, more recent evidence suggests that environmental factors contribute to the development of chronic noncommunicable disease (143). Environmental stressors such as noise and air pollution are becoming more and more important in our industrialized world and especially traffic noise from road, aircraft, and railway transportation represents a potential novel cardiovascular risk factor (162, 166), and numerous studies demonstrate that noise plays a role for the development of cardiovascular as well as metabolic disease (167). Since there are almost no models for translational research in humans and animals, the detailed mechanisms responsible for noise-triggered cardiovascular disease (CVD) are still elusive. Current concepts are based on chronic stress reactions such as activation of the autonomic and endocrine system caused by annoyance or sleep deprivation leading to subsequent pathophysiologic systemic alterations, all of which contribute to the progression of CVD (14, 16).

**FIG. 1.**
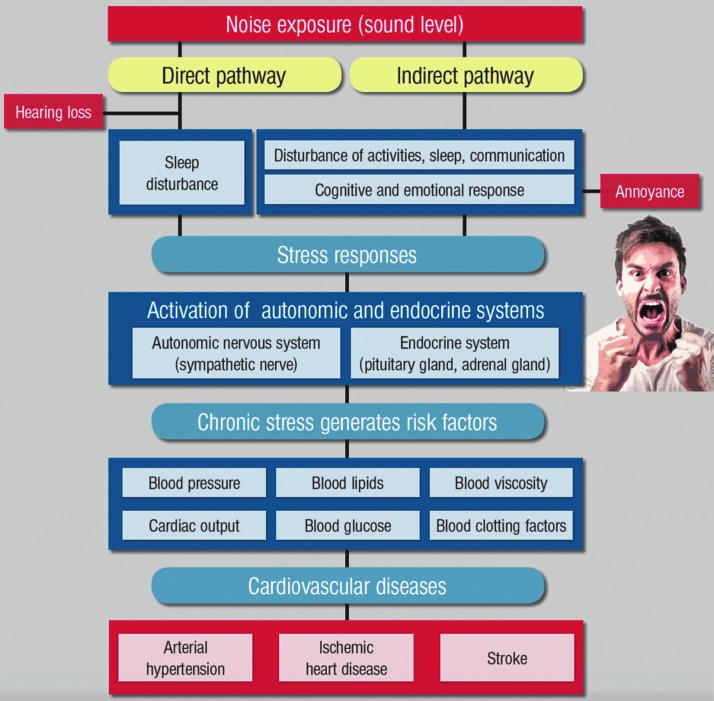
**Noise reaction scheme explaining the adverse cardiovascular effects of noise exposure.** Adapted from Münzel *et al*. (162) with permission of the publisher. Copyright © 2014, Oxford University Press.

Babisch established the modern noise reaction model, postulating an “indirect pathway,” in which disturbance of sleep, communication, and activity by low-level noise exposure causes changes of emotional and cognitive parameters and annoyance, followed by chronic stress reactions and adverse health effects (13, 14) (summarized in Fig. 1). Importantly, environmental stressors generate their own cardiovascular risk factors such as hypertension, hyperglycemia, hyperlipidemia, and increased blood viscosity and coagulation (13), contributing to CVD such as coronary artery disease, heart failure, and stroke. A case report on Takotsubo syndrome in a patient attributed this cardiomyopathy that is linked to excessive stress hormone release to annoyance in response to exposure to nighttime aircraft noise exposure (164). The degree of noise-induced annoyance determines the effect of the noise level on arterial hypertension (21) and ischemic coronary artery disease (14). As shown by community-based studies, high levels of environmental noise lead to mental health symptoms (*e.g.*, depression and anxiety) and the degree of noise annoyance may be directly associated with future development of depression and anxiety disorders (31), all of which have negative effects on cardiovascular function (220).

The molecular mechanisms of noise-triggered vascular damage and induction of CVD are still elusive. Chronic stress reactions and increases in circulating cortisol levels (see section III.C.5) (13, 14, 16, 18) are thought to induce vascular oxidative stress with subsequent (endothelial) dysfunction (207, 208) and prothrombotic and inflammatory pathways (43). Of note, noise-induced cardiovascular damage is a multifactorial process and the different pathomechanisms may be active at differing time points of noise exposure (167).

### A. Noise and global burden of disease

Over the last two decades, there was a substantial shift of the major risk factors that contribute to the global disease burden. In the year 1990, the most important risk factors were communicable childhood diseases, whereas in the year 2010, they were mostly replaced by those comprising noncommunicable adulthood diseases, also reflecting the aging population in the Western societies (143, 168). Besides the demographic changes, the clinical improvement of the treatment of childhood diseases, and prenatal mortality, also advances in the quality of drinking water, nutrition, sanitary conditions, and indoor air pollution are the main reasons for this shift of leading risk factors for global deaths and diseases within the last two decades [for review, see Daiber *et al.* (57)]. Of course this shift is not equally visible in all regions of the world since the socioeconomic status of different territories may still promote poverty and communicable childhood diseases as the most important risk factors of death and severe illness (*e.g.*, in sub-Saharan Africa).

**FIG. 2.**
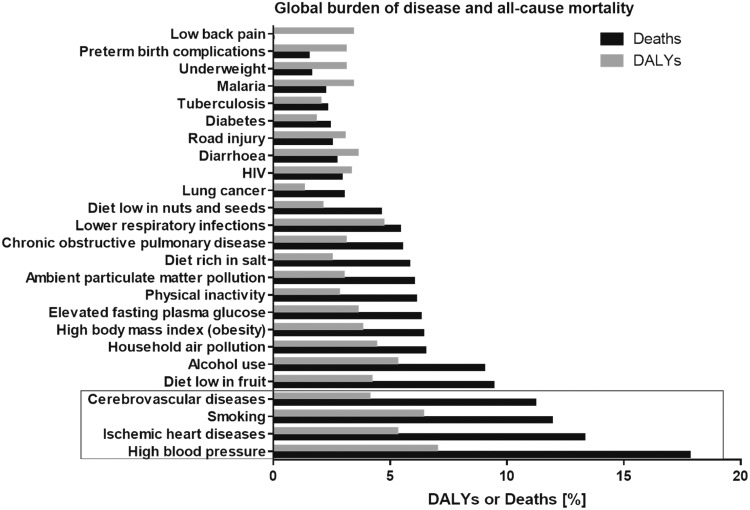
**The most important diseases/injuries and risk factors for the global all-cause mortality and life years spent with significant illness and/or disability.** DALYs, disability-adjusted life years. Figure was drawn *de novo* according to data presented by Lim *et al.* (143) and Murray *et al.* (168).

According to a previous meta-analysis of multiple clinical studies on the global all-cause disease burden and mortality, cardiovascular risk factors (arterial hypertension and smoking) and diseases (ischemic heart disease and cerebrovascular disease) represent the top four causes of death and reduced life quality due to illness (disability-adjusted life years, or DALYs) in humans worldwide (Fig. 2) (143, 168). Among those, high blood pressure (BP) is the leading risk factor for all-cause mortality and has the most pronounced impact on life years spent with significant illness and disability of the global population. Based on these observations, CVDs have outcompeted underweight, water pollution, low hygienic standards, air pollution by household heating and fire places, infectious diseases, as well as early childhood diseases, as the number one cause of global deaths within the last 20 years (143, 168).

**FIG. 3.**
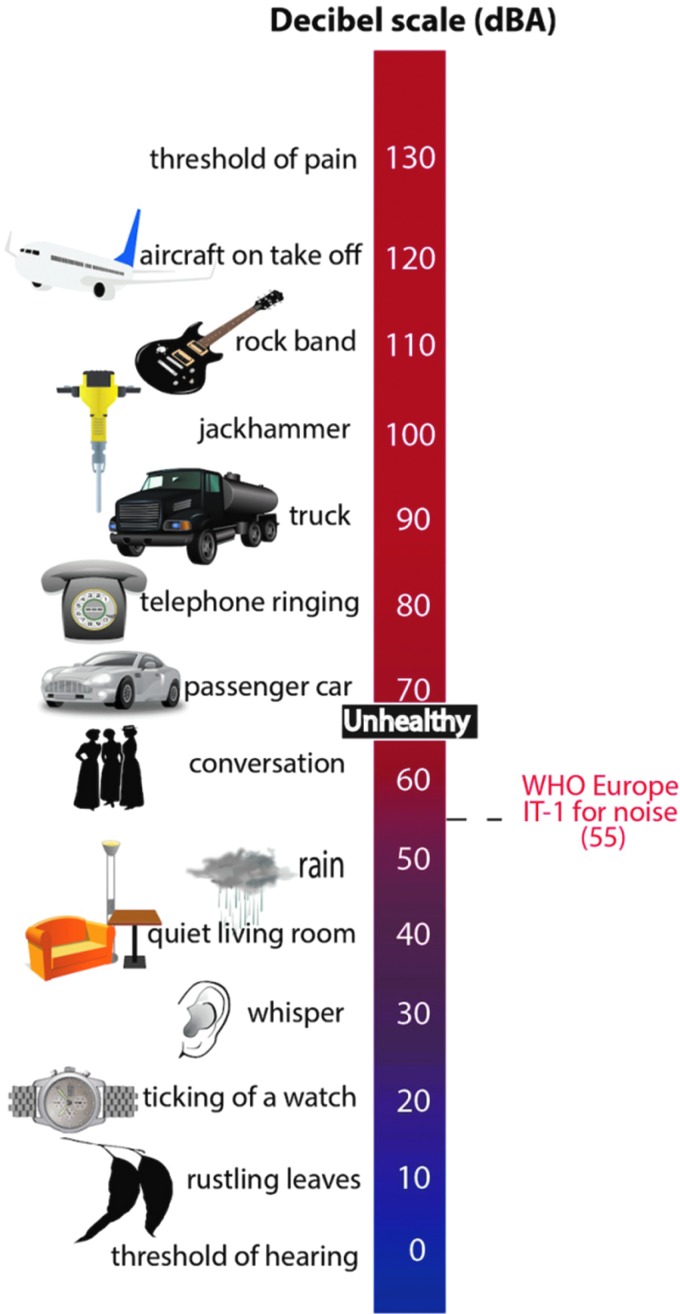
**Noise thresholds and guidelines.** Adapted from Münzel *et al.* (166) with permission of the publisher. Copyright © 2017, Oxford University Press.

The nonauditory effects of noise comprising annoyance, sleep disturbance, and psychological stress are believed to cause global disability. It is estimated by the World Health Organization (WHO) that in Western Europe a total number of 1,685,000 DALYs are lost for ischemic heart disease, cognitive impairment of children, sleep disturbance, tinnitus, and annoyance every year (2). This means that traffic noise exposure accounts for the loss of more than 1,000,000 healthy life years in the Western European population every year. Sleep disturbance and annoyance induced by exposure to road traffic noise are responsible for the majority of environmental noise-related diseases in Western Europe. It is estimated that 40% of the European population is exposed to noise originating from road traffic at levels exceeding 55 A-weight decibels [dB(A)]; 20% exposed to levels exceeding 65 dB(A) during the daytime; and 30% of the population is exposed to levels exceeding 55 dB(A) at night (2). Sound levels of different noise sources are provided in Figure 3. Asian populations living in urbanized territories may face much higher traffic noise exposures compared with European cities, even reaching L_den_ levels (Day–Evening–Night level, *i.e.*, the average sound pressure level (L_eq_) measured over a 24-h period, Table 1) of 60–64 dB(A) or more (138).

**Table 1.** Definitions for Noise-Specific Research

L_Aeq16_: Indicates noise exposure over a 16-h daytime period usually from 7 to 23.00. The same time period is sometimes represented by L_day_, which indicates noise exposure over a 12-h daytime period.
L_night_: Noise exposure between 11 pm and 7 am.
L_Aeq8h:_ Noise exposure over an 8-h nighttime period.
L_DN_: The average equivalent sound level over a 24-h period with a 10 dB penalty for noise recorded between 11 pm and 7 am. Like L_DEN_, L_DN_ calculator accepts hourly L_eq_ measurements and calculates the L_DN_ accordingly.
L_DEN_: The 24-h L_eq_ calculated for an annual period, but with a 5 dB penalty for evening and a 10 dB penalty for night. The penalties are introduced to indicate people's extra sensitivity to noise during the evening and the night. With respect to long-term health effects, these metrics are calculated as average annual exposure indicators.
L_max_: Maximum noise level in a given time period. L_max_ is often better at predicting acute effects of single noise events than average noise levels. With respect to long-term health effects, however, the integrated sound level over a long period of time seems more appropriate for a description of the noise.

The energy-equivalent average A-weighted sound pressure level (L_Aeq_) as expressed in decibels (dB) is the most commonly used indicator of the noise exposure that people perceive outside and inside their homes. The A-weighting accounts for the different sensitivities of the human ear at different sound frequencies. L_eq_, average sound pressure level. Adapted from Münzel *et al.* (162) with permission of the publisher. Copyright © 2014, Oxford University Press.

A critical point when conducting studies of noise and health is adjustment for confounders. Traffic noise is known to negatively impact property prices (250), which highlights the need for proper adjustment for socioeconomic status (SES) and lifestyle in studies of traffic noise and health. Cohort studies often have the most detailed set of confounders in the analyses, including both SES variables and lifestyle habits (216, 222). Recently, a number of large population-based studies (>750,000 participants) on noise and health have been published, which, due to their register-based nature, lack information on lifestyle risk factors (100, 108, 214). These studies normally include some adjustment for SES: while some studies have detailed information on a personal level of, for example, income and education (108), others rely mainly on a more crude area-level information of SES (100, 214). It is, however, important to note that adjusting for lifestyle in studies of noise is not straightforward, as a number of studies have linked traffic noise with, for example, obesity and physical activity (49, 68, 84, 189, 200), strongly suggesting that these are intermediates and not confounders on the pathway between traffic noise and disease.

### B. Historical view on noise research: the concept of nonauditory effects of noise

Being a visionary, Robert Koch postulated in 1910 that “One day mankind will have to fight the burden of noise as relentless as the pest and cholera” (1). This postulate was confirmed by the WHO in 1995 stating that “The main negative effects of such noise on people are disturbances of communication, rest and sleep, and general annoyance. Over long periods of time these effects have a detrimental influence on wellbeing and perceived quality of life.” (2).

**FIG. 4.**
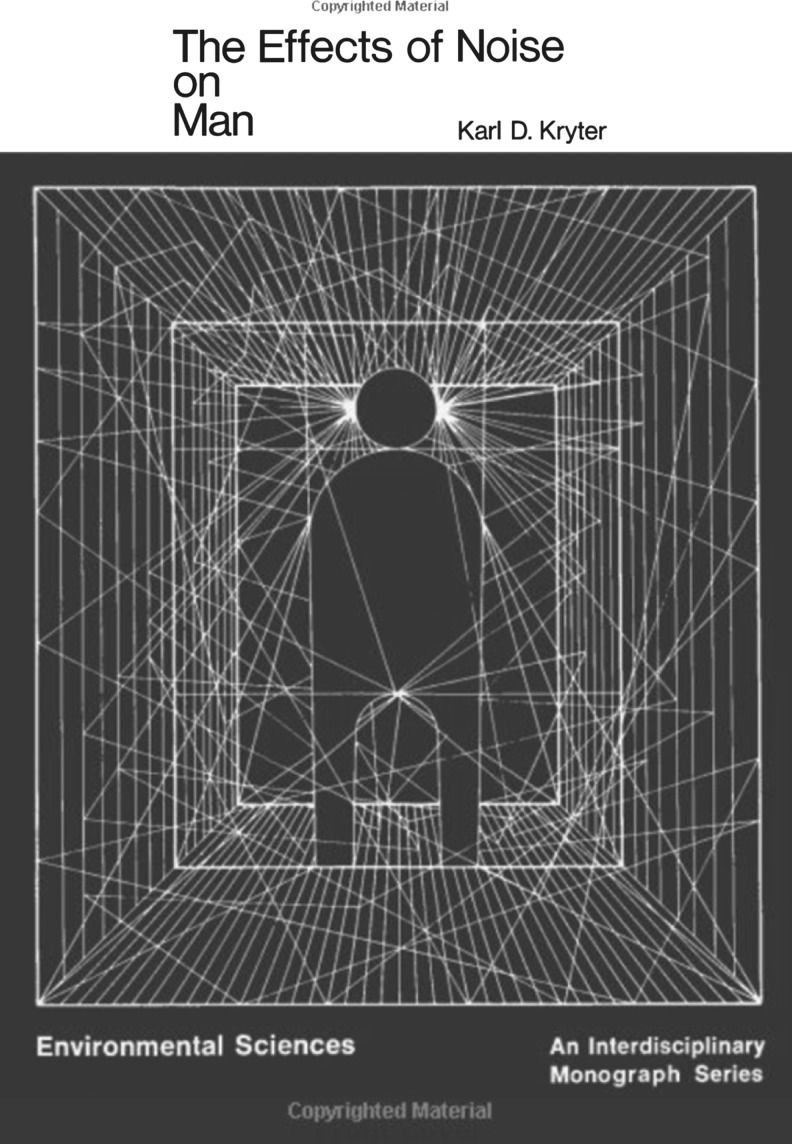
**Title page of the Book from Karl Kryter summarizing the effects of noise on man, discussing man's nonauditory system responses including information about the effects of noise on work performance, sleep, feelings of pain, vision, and blood circulation.** Book cover art by Kryter (130). Copyright © 1970 Academic Press, Inc. Published by Elsevier Inc. All rights reserved.

The concept that the so-called nonauditory effects contribute substantially to health consequences was already mentioned in the monography named “Effects of Noise in Man,” by Karl D. Kryter (Fig. 4). In his book, he summarized the nonauditory systemic responses, including information about the effects of noise on work performance, sleep, feeling pain, vision, and blood circulation. The monography was started in 1950 and finally published in 1970 (130). Importantly, Kryter states that some of the more complex and perhaps more important (from a health viewpoint) effects of noise have to do with these somewhat second-order reactions. He proposed that the nonauditory effects are the result of the stimulation of three neural systems that are not exclusively linked to audition: (i) the autonomic nervous system that controls systemic somatic responses and arousal reactions of the organism—the glands, the viscera, and circulatory system. (ii) The reticular nervous system, leading to arousal responses of the central nervous system as well as organs of perception related to pain and pleasure. (iii) The cortical and subcortical brain centers responsible for intellectual performance.

In the chapter “General Physiological Responses to Noise,” Kryter cites articles dealing with observations that acute noise exposure has cardiovascular effects. Jansen showed in 1964 that noise exposure in subjects performing exercise is causing vasoconstriction (116). Furthermore, Jansen and Klensch also reported considerable variations in the hemodynamic responses of subjects exposed to noise or music. By measuring blood circulatory responses to a noise of 90 phon, the authors demonstrated that responses are not uniform (118). While the majority of the noise- or music-exposed subjects showed a decreased cardiac output and minute flow during the noise or music, some behaved otherwise. The authors also concluded that the similarity of responses to noise or music may indicate that the intensity of sound and not its aversive (noise) or its pleasurable (music) aspects controlled the somatic responses. Thus, it may be possible that the music was stressful mainly because of its level (118).

**FIG. 5.**
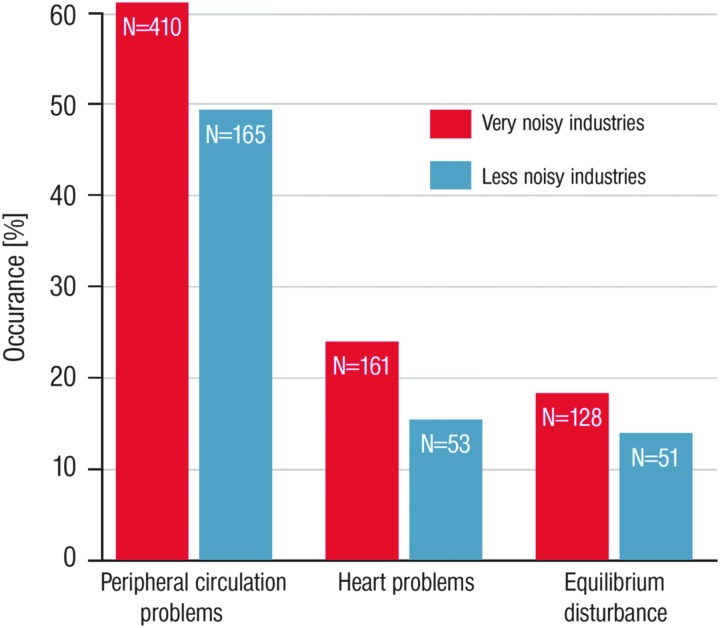
**Difference in percent of occurrence of physiological problems in 1005 German industrial workers.** Figure drawn *de novo* from data reported in Kryter (131).

That noise is causing stress was also demonstrated by Levi (141). He showed that work in industrial and office environments leads to increased excretion of catecholamines as a marker of increased stress. Evidence that noise may cause cardiovascular problems was provided by Jansen (117). He reported the occurrence of physiological problems in 1005 German industrial workers such as peripheral circulation problems, heart problems, and equilibrium disturbances, which were more pronounced in very noisy industries compared with less noisy industries (Fig. 5). Thus, based on these early observations, there was no doubt that chronic noise exposure may cause CVD.

Experimental studies have shown that noise may lead to release of stress hormones [for review, see Noise in Europe 2014 (14)]. This was observed in studies on aircraft noise (148, 149) and road traffic noise exposure (113, 115). Interestingly, music has also been shown to increase catecholamine and cortisol levels (30). In addition, intermittent noise caused stronger effects on norepinephrine and corticosteroid levels than steady noise (275, 276). Similar effects were observed for low-frequency noise (114). Thus, with substantial discoveries on stress response pathways in the early 90s, research on the adverse effects of noise on the nonauditory system envisaged a breakthrough (231), and the adverse effects of noise exposure on health were more and more perceived as a “neuroendocrine complication” (34, 127, 150).

A search through the noise literature retrieved 145 reviews on noise and human health published since the year 1980, including 43 reviews from gray literature (*e.g.*, materials and research produced by organizations outside of the traditional commercial and academic publishing systems and distribution channels such as research summaries published on university home pages, private and charitable foundations, or by authorities such as the European Commission, WHO, or German Government). The list of these reviews can be found in ENNAH—European Network on Noise and Health (3). A quality assessment of the peer-reviewed journal articles by the European Network on Noise and Health (ENNAH) researchers showed that not more than 10 articles were of high quality, while 9 and 83 articles were classified as medium and low quality, respectively. Most of the review articles were of narrative type with lower scientific impact than systematic reviews and meta-analyses. For risk assessment, high-quality meta-analyses that contain details on dose/response are preferred [*e.g.*, see Babisch (17), van Kempen and Babisch (255), van Kempen *et al.* (256), and Vienneau *et al.* (261)].

From the early 60s, human laboratory studies on the nonauditory effects of noise exposure were routinely conducted. However, only short-term noise exposure was studied in these setups, which cannot reflect the chronic effects of environmental noise exposure [numerous examples are provided in the WHO and JRC Report (2)]. From the year 1990, many human studies were conducted for work safety reasons. Occupational studies provided evidence for health disorders in workers chronically exposed to noise for several years, although noise exposure levels among these workers were much higher than in the ambient environment [several examples are provided in the WHO and JRC Report (2)].

Animal models were routinely used since the 60s with a few examples of studies on noise exposure effects on cardiovascular function [numerous examples are provided in the WHO and JRC Report (2)]. BP increases in response to noise were studied in chronically exposed monkeys (185) or rats (7). More examples on noise effects on collagen in the rat heart, platelet adhesiveness, insulin secretion, or animal health in general can be found in the WHO and JRC Report (2). Animal studies on noise effects on cardiovascular function (besides BP) are rare, especially those using reasonable sound pressure levels (SPLs) below 100 dB(A). It may be argued that comparison of the effects in human subjects and animals is questionable, particularly since two response routes are operative: (i) the direct effect due to nervous innervation; (ii) the indirect effect due to the cognition of the sound, which should be completely different in animals and humans.

A large part of previous large-scale animal and human studies on noise-dependent effects on physiological functions and health were conducted for military reasons, for example, to study the effects of low-altitude flights of military jets on these parameters in humans of inhabited regions and also in farm animals. The latter studies were, in most cases, rather focused on the productivity of the farm animals (*e.g.*, effect of low-altitude flights of military jets on milk production of dairy cows or on egg production of layers) than on the health status of these animals. Another part of large-scale animal studies on noise-dependent effects on behavior were conducted in wild animals such as deer, boar, and birds to learn more about the impact of traffic noise on deer crossing and flyways. Most of the latter studies were commissioned for effects of noise on birds by the aviation regulating authority. Only very few of these previous studies assessed redox biomarkers and characterized the role of oxidative stress for noise-dependent effects on health, although recent reports highlight that oxidative stress and redox regulation largely affect gene regulation, stress adaptation processes, and other essential biological pathways (65, 157).

### C. Impact of environmental noise on healthcare systems

Although environmental noise is not yet fully accepted as a cardiovascular risk factor, public health authorities and organizations are alarmed by the striking emerging evidence for the adverse cardiovascular effects of environmental noise exposure. The ENNAH project funded by the European Union's (EU) Seventh Framework Program highlighted in the final report the alarming epidemiological evidence for an increased incidence of CVD due to environmental noise exposure (3). The ENNAH Network comprised 33 European research centers from 16 countries to define future research directions and policy needs for the impact of noise on health in Europe. The research focus of ENNAH was to study environmental noise sources, in particular transportation noise. The tasks and merits of the ENNAH Network also comprised the identification of gaps in the research on noise effects on health as well as assessment, prioritization, and integration of the future research directions into the development of strategies for a more efficient noise research funding. The generation of noise maps under the Environmental Noise Directive (2002/49/EC) emerged as a highly efficient tool for noise and health research although comprising major advantages and disadvantages that, however, could be overcome by the implementation of new methods for acoustic measurement and modeling to be used in future noise and health studies.

Another major output of ENNAH research was its contribution to the important publications from the WHO and European Commision's Joint Research Centre (JRC) on the “Burden of Disease from Environmental Noise.” As already outlined above, the WHO estimates that traffic-related noise is responsible for the loss of at least 1,000,000 healthy life years every year in Western European countries. Road traffic is the most dominant source of environmental noise with 125 million people being affected, followed by rail traffic noise (nearly 8 Mio.) and aircraft noise (almost 3 Mio.) (2). The “cardiovascular burden” of noise is substantial in Europe, reflected by 900,000 cases of hypertension, 43,000 hospital admissions, and more than 10,000 premature deaths per year related to coronary heart disease and stroke, all related to noise-triggered adverse effects (4). There is substantial evidence for adverse effects of environmental noise on health that are associated with higher incidence of CVD, increased BP, mental stress responses such as annoyance or sleep disturbance as well as psychological disorders and intellectual performance.

In a recent CE Delft report (60), it was speculated that noise originating from railway and road traffic accounts for up to 50,000 fatal heart attacks and 245,000 cases of ischemic heart disease every year in the EU25 member countries. The probability of heart disease was estimated of the annual number of people experiencing a fatal heart attack in association with traffic noise published in Babisch (15) [for official reports, see ENNAH—European Network on Noise and Health (3)] considering the part of population with noise exposure levels over 60 dB(A) in the respective countries. The total expenses to our societies, inflicted by traffic noise exposure in the EU27 (except Cyprus, Estonia, Latvia, Lithuania, and Malta) member countries, also considering charges to health services, may reach 40 billion EUR per year [for official report, see ENNAH—European Network on Noise and Health (3)].

According to a study in the United Kingdom, noise levels of ≥55 dB(A) during daytime may account for an additional 542 patients with hypertension-related myocardial infarction, 788 patients with stroke, and 1169 patients with dementia, leading to additional expenses of approximately £1.09 billion per year (103). A recent economic assessment in the United States of environmental noise as a cardiovascular risk factor revealed similar economic burden for noise-related disease (243). The results revealed that a 5 dB(A) noise reduction scenario would reduce the prevalence of arterial hypertension by 1.4% and coronary heart disease by 1.8%. The economic benefit calculated would reach an estimated 3.9 billion dollars.

A comparative health risk assessment for Switzerland in 2010 revealed additional costs of 1050 million Swiss Francs (CHF) related to diminished prices for houses and apartments (rental and purchase) in Switzerland for the year 2010 (260). The impact of traffic-dependent noise exposure on CVDs was 560 million CHF based on lost life years (including DALY) and 190 million CHF related to noise-associated morbidity. This amounts to total additional expenses in the health system that are related to noise exposure of 1800 million CHF, which is comparable to the total air pollution-associated additional costs of 1760 million CHF (1250 million CHF related to lost life years and 510 million CHF associated with noise-induced morbidity).

Overall, these studies clearly show that environmental noise exposure affects public health with measurable medical and economic implications. Thus, there is an urgent need to get a pathophysiological insight into the mechanisms being responsible for noise-triggered CVD, which may help to successfully implement mitigation strategies and successful specific pharmacotherapy.

## II. Epidemiology: Traffic Noise and Cardiometabolic Disease

According to the majority of studies on chronic exposure to transportation noise (road, railway, and/or aircraft traffic), there is a significant association between higher BP, hypertension or the prescription of antihypertensives, ischemic heart disease (*e.g.*, myocardial infarction), cerebrovascular disease (*e.g.*, stroke), neuronal disorders (*e.g.*, dementia), and cardiometabolic disease (*e.g.*, diabetes mellitus).

### A. Cardiovascular disease

Epidemiological research on traffic noise and health has, during the last decades, focused on cardiovascular effects, especially elevated BP and ischemic heart disease. In 2012, a meta-analysis, including 24 cross-sectional studies of road traffic noise and hypertension, found a 3.4% higher probability of prevalent hypertension per 5 dB higher road traffic noise (odds ratio: 1.034; 95% confidence interval [CI]: 1.011–1.056) (255). This result has been confirmed in later studies also including adjustment for air pollution (85, 225). Most of these studies relied on estimation of outdoor noise as a proxy for exposure. Interestingly, a Spanish study estimated exposure to nighttime noise, both outdoors and indoors (using information on *e.g.*, orientation of bedroom and indoor insulation), and found that indoor nighttime noise levels were more often associated with elevated systolic BP and hypertension than the outdoor nighttime noise levels (85).

**FIG. 6.**
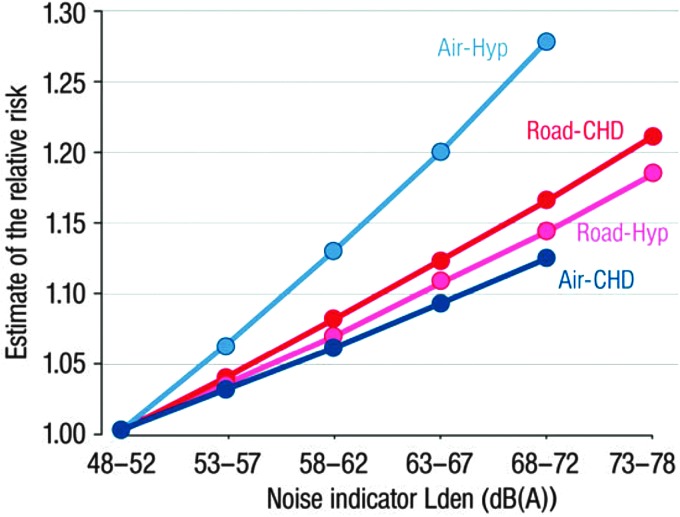
**Exposure–response relationships of the associations between transportation noise and cardiovascular health outcomes.** Air, aircraft noise; dB(A), A-weight decibels; CHD, coronary heart disease; Hyp, hypertension; Road, road traffic noise. Adapted from Münzel *et al.* (166) with permission of the publisher. Copyright © 2017, Oxford University Press.

Studies have also indicated an association between exposure to aircraft transportation noise and hypertension (69, 74, 119, 199). The Hypertension and Exposure to Noise near Airports (HYENA) study is one of most comprehensive of these studies, and based on almost 5000 study participants from 6 European countries (119). This study found that a rise in nighttime aircraft transportation noise of 10 dB(A) is associated with an odds ratio of prevalent hypertension of 1.14 (95% CI: 1.01–1.29) (Fig. 6).

The above-described studies are cross-sectional, which prevent conclusions on causality. Studies on noise and incident hypertension are, however, emerging for both road traffic and aircraft noise, and they largely show that traffic noise also increases risk for hypertension, thereby supporting the cross-sectional findings (61, 69, 88).

Several studies have addressed the association between traffic noise exposure and ischemic heart disease; many of high quality. These studies have been included in two recent meta-analyses (17, 261). In 2014, Babisch investigated the relationship between the exposure to road traffic noise and coronary heart disease and found a pooled estimate of 1.08 (95% CI: 1.04–1.13) per 10 dB(A) higher exposure (17). A similar result was obtained by Vienneau *et al.* in 2015 who included studies of both road traffic and aircraft noise (261). The pooled estimates in this meta-analysis were 1.06 (95% CI: 1.03–1.09) per 10 dB(A) increase in noise exposure (Fig. 6). Importantly, in four of the studies, the association between transportation noise exposure and ischemic heart disease was not attenuated on adjustment for air pollution as a confounder. The authors of both meta-analyses concluded that exposure to transportation noise is a significant cardiovascular risk factor.

Recent studies have found traffic noise to increase the risk for another major CVD, namely stroke (98, 100, 224). Three large studies have been conducted: two on road traffic noise (98, 224) and one on aircraft noise (100). The first study from 2011 was based on a large Danish cohort and found that exposure to road traffic noise at home increased risk for stroke by 14% per 10 dB(A) rise in noise (95% CI: 1.03–1.25). A later study using the same cohort found the effect of traffic noise on stroke to be confined to ischemic strokes (227). These results were in 2015 confirmed by a study covering all of London, which showed a relative risk of 1.09 for stroke (95% CI: 1.04–1.14) in the older population, when comparing people exposed to more than 60 dB(A) with those exposed to <55 dB(A) (98). Finally, a study of airport noise around Heathrow in London showed similar results: a relative risk of 1.24 (95% CI: 1.08–1.43) when comparing highly exposed people [>63 dB(A)] with people exposed to 51 dB(A) or less (100).

### B. Metabolic disease

Recent research has shown that transportation noise exposure may lead to obesity and increase risk for type 2 diabetes mellitus (48–50, 68, 177, 189, 222). The suggested mechanisms include an effect of noise-induced stress and disturbance of sleep on appetite regulation, alterations in the glucose regulation, decreased levels of insulin, and reduced insulin sensitivity (37, 39, 229, 230, 233, 244, 247). However, induction of oxidative stress is regarded a critical factor in the pathogenesis of diabetes mellitus, and in light of the emerging evidence on noise and oxidative stress (161, 208), it seems likely that oxidative stress also plays a role in the pathway between noise and diabetes.

Recent studies from the Nordic countries have examined the association of traffic noise, from roads, railways, and aircrafts, with obesity among cohorts of adults (49, 50, 68, 177). Three of these studies were cross sectional and based on cohorts ranging from 5000 to 57,000 participants. While two of the studies revealed that exposure to road, railway, or aircraft noise was statistically significantly associated with mainly an increased waist circumference (50, 177), the third study found no overall association (177). However, two prospective studies have also been conducted, and both studies found an association of noise exposure with a statistically significant gain in waist circumference (49, 68), supporting the concept that traffic noise exposure leads to cortisol release, a well-known trigger of central obesity. Finally, a Danish study of a cohort of 40,000 children indicated that exposure to noise from road traffic may be positively associated with odds for childhood obesity (48).

In 2013, a large Danish cohort study addressed the relationship between road traffic noise and risk for diabetes, based on a prospective cohort of 57,000 persons of whom almost 4000 developed diabetes during follow-up (222). The study found that a 10 dB rise in long-term exposure to residential noise from road traffic was associated with an 11% significantly increased risk for diabetes, even after adjusting for several confounders (*e.g.*, air pollution). These results were recently confirmed in a Swiss study based on a cohort study of 2631 persons (75). This study found that noise from road and aircraft traffic is associated with incident diabetes, with relative risks of 1.35 (95% CI: 1.02–1.78) for road traffic noise and 1.86 (95% CI: 0.96–3.59) for aircraft noise per interquartile range increase in noise, after adjusting for air pollution and other confounders. Interestingly, the study found more pronounced effects of road traffic noise among people who reported lower sleep quality or who sleep with windows open (75).

Importantly, obesity, metabolic syndromes, or diabetes are linked to endothelial dysfunction and increased oxidative stress within the vessels, providing evidence that the prodiabetic effects of noise may lead to similar vascular phenomena [for review, see Gori and Münzel (95) and Münzel *et al.* (163)].

### C. Cancer

Only a few studies have investigated whether traffic noise exposure is associated with a higher incidence of cancer. However, recent findings suggesting that exposure to noise potentially leads to oxidative stress (208) make investigations of the effect of noise on risk of cancer very relevant. Currently, three studies have addressed the relationship between noise and cancer in a Danish cohort of 30,000 women and 27,000 men (201, 202, 226). Exposure to noise from road and railway traffic displayed a significant association with risk for estrogen receptor-negative breast cancer (226). Supporting this result was the finding that both road traffic noise and railway noise—two noise exposures that were not correlated—increased the risk for estrogen receptor-negative breast cancer independently of each other. However, the study included only 203 women with estrogen receptor-negative breast cancer. These results have later been partly supported by a large German study that found an association between aircraft noise and higher risk of estrogen receptor-negative breast cancer, although no associations were found for road and railway noise (105). In an investigation of the association between traffic noise and colorectal cancer incidence in the Danish cohort, an association with borderline significance was observed between road traffic noise and colorectal cancer, which was mainly restricted to distal colon cancer (202). Finally, investigations in the same cohort revealed no association between noise exposure and prostate cancer (201).

### D. Effects of noise exposure on sleep: Short sleep, endothelial dysfunction, and oxidative stress

Lack of sleep caused by sleep restriction or sleep fragmentation has been demonstrated to exert detrimental effects on different systems, including alterations of metabolic, endocrine, and immune signaling cascades. Acute and chronic sleep restriction and/or fragmentation cause inadequate insulin secretion, decreased insulin sensitivity, increased sympathetic tone, and arterial and venous endothelial dysfunction [for review, see Cappuccio *et al.* (38)]. Epidemiological studies also provided evidence that short sleep, <6 h/night, is associated with cardiometabolic diseases such as obesity, diabetes mellitus, arterial hypertension, and increased all-cause mortality (38), highlighting the important role of sleep disruptions and insufficient length for cardiovascular health. Thus, sleep deprivation and fragmentation are regarded the most important nonauditory effects of environmental noise exposure. Changes in the sleep architecture induced by noise cause modification of sleep stages, induce frequent arousals, and increase the duration of frequent awakenings. Traffic noise during sleep was also related to autonomic arousals and increased heart rate (96).

Two hours of sleep deprivation for 8 days cause endothelial dysfunction in healthy subjects, and the degree of deterioration of endothelial function is comparable to that observed in workers working 24-h shifts (9) and in humans exposed to chronic sleep restriction (245). Thus, a likely explanation for the development of endothelial dysfunction caused by sleep-phase noise exposure may be sleep deprivation and fragmentation, conditions linked with increased cardiovascular events and mortality (46, 78).

In a mouse model, Carreras *et al.* demonstrated that endothelial dysfunction and arterial hypertension develop on exposure to 20 weeks of sleep deprivation (40). The authors observed a marked disruption of vascular elastic fibers and an increase in foam cells and macrophages within the vascular wall. In addition, sleep fragmentation reduced messenger RNA (mRNA) expression of the senescence markers TERT and cyclin A, the tumor suppressor p16^INK4^, and interleukin-6 (IL-6) levels (40). The same group also showed that sleep fragmentation causes activation of NADPH oxidase in the brain (169) and increases oxidative stress induced by the NADPH oxidase subunit NOX2 within adipose tissue (203), all of which were associated with insulin resistance. Sleep disturbance and deprivation even gain more importance when considering the fact that essential vascular repair takes place during the sleep time, which is in line with impaired stroke recovery of rats subjected to sleep disturbances (283), that awakening affects the circadian clock with impact on vascular function and regeneration (5), and that the circadian clock *per se* is subject to redox regulation (269).

### E. Traffic noise exposure, annoyance, noise sensitivity, and mental disease

Annoyance is a widely observed response to environmental noise in the population (2), originating from negative effects on daily activities, feelings, thoughts, sleep, or rest, and can also comprise negative emotions, such as irritability, distress, exhaustion, and other stress responses (180). Severe annoyance can be linked to decreased well-being and health, and due to the impact on a large population, there is a significant contribution of annoyance to the burden of disease from exposure to environmental noise. While noise and annoyance are clearly correlated, the noise responsiveness and sensitivity of each individual are determined by individual factors, including genetic and physiological states as well as life style. Of note, repeated aircraft noise exposures were associated with a measurable augmentation of annoyance reactions in the longer run and annoyance ratings according to the HYENA study exceeded the estimations by the EU standard curves (19).

Individuals with substantial annoyance responses had higher incidence of mental and physical symptoms and higher prescription of psychotropic drugs and more frequent general practice and outpatient services (264). As reviewed by van Kamp and Davies (254), subjects with mental disorders have a higher noise sensitivity that is comparable to people with chronical somatic illness or suffering from tinnitus as well as shift workers, fetuses, and neonates, which may translate to more frequent adverse effects of noise on health. Preliminary studies revealed qualitative differences in the effects of specific traffic noise sources on annoyance responses (211, 235).

**FIG. 7.**
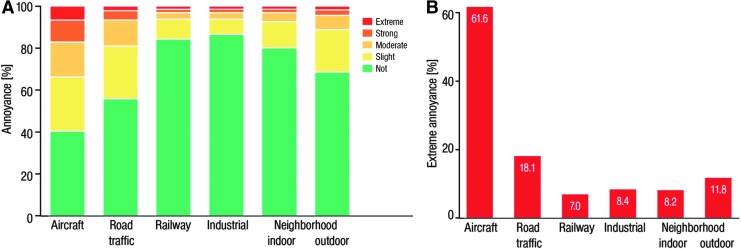
**Relationship between environmental noise sources and the degree of annoyance.** **(A)** Degrees of overall annoyance according to different sources of noise. **(B)** Sources of extreme annoyance. Adapted from Beutel *et al.* (31) with permission of the publisher/authors. Copyright: © 2016, Beutel *et al.* (open access).

Aircraft noise was generally considered more annoying, displaying a more pronounced impact on sleep, than noise exposure from road and railway traffic (67, 156). A recent study of 15,010 subjects (selected randomly from the local registry in the city of Mainz and the district of Mainz-Bingen, a large population-based, prospective, observational, single-center cohort study in the Rhine-Main-Region in Western Mid-Germany) revealed that aircraft noise exposure was a major environmental trigger of public annoyance in 60% of the included individuals (Fig. 7A, B) (268).

**FIG. 8.**
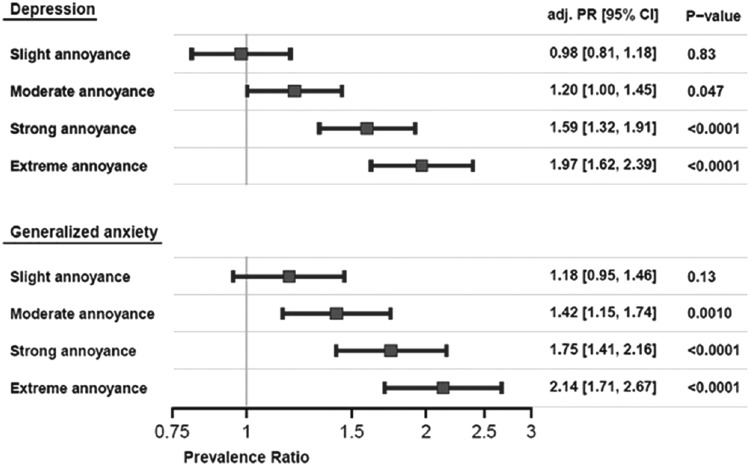
**Association between noise annoyance, depression, and anxiety.** CI, confidence interval. Adapted from Beutel *et al.* (31) with permission of the publisher/authors. Copyright: © 2016, Beutel *et al.* (open access).

Most included subjects were obviously annoyed by noise exposure; only 20.7% were not annoyed. More than half (52.8%) of the people reported at least moderate annoyance. The study results also revealed an association of annoyance by noise exposure with depression and anxiety, even when adjusting for the confounding factors, sex, age, and SES (Fig. 8) (31). Compared to no annoyance, the odds ratio for depression showed a continuous increase starting from no or slight annoyance, over moderate and strong to extreme annoyance, the latter displaying a 2.12-fold increase in the incidence of depression. Similarly, the probability of anxiety showed a steady increase from no annoyance, over slight, moderate, and strong to extreme annoyance (2.28-fold) (31).

Recently, Seidler *et al.* showed a significant association of exposure to traffic noise with depression (213). The relationship between exposure to road traffic noise and the risk of depression was linear with an odds ratio of 1.17 for a 24-h sound level >70 dB(A). The relationship between exposure to aircraft noise and the risk of depression revealed an odds ratio of 1.23 at quite low sound levels of 50–55 dB(A), interestingly, the risk estimates decreased at higher sound exposure levels, most probably since noise-sensitive people escape the exposure (*e.g.*, by moving to another place). For noise from railway transportation, the odds ratio peaked at 60–65 dB(A). Importantly, the highest odds ratio of 1.42 was found in that part of the study population that was exposed to combined noise from all three sources at sound levels above 50 dB(A).

In contrast to annoyance, noise sensitivity is a stable response to noise and has been demonstrated to be an independent predictor of the annoyance to environmental noise (254). Accordingly, it has been proposed that noise sensitivity might be an indicator of vulnerability to environmental stressors, meaning that highly noise-sensitive people will develop more diseases in response to environmental noise (236). Recent results in 3630 male and female civil servants from the U.K. Whitehall II study demonstrated that noise sensitivity and CVD morbidity or mortality were not associated, except in people with lower employment grades, where the authors established an association with angina pectoris (238). Being highly sensitive to noise was more common in 50–55-year-old women, and those of high employment grade. Furthermore, noise sensitivity was a constant predictor of depressive symptoms and psychological distress. The authors concluded that noise sensitivity has been identified as a predictor of mental illness (238).

### F. Effects of noise and air pollution coexposure

Most studies on air pollution do not pay attention to noise levels, and likewise, the majority of noise exposure studies do not include measures for air pollution. A rather small number of studies addressed both environmental risk factors, air pollution and traffic noise, and adjusted for the respective confounding pollutant in the overall statistical models (51, 59, 82, 98, 216, 223, 227). A majority of these studies suggest that traffic noise and air pollution are independent risk factors of CVD incidence and mortality. A meta-analysis from 2013 found that adjustment for one of these confounders, air or traffic noise pollution, caused no loss of their association with CVD outcome (249). However, also the opposite effect was observed in some studies, meaning that the association of road traffic noise or air pollution with CVD was lost after adjustment for the complementary risk factor, respectively (82, 227).

Obviously, the interpretation of results of studies on road traffic noise and air pollution is complicated by the appreciable colinearity of these two environmental risk factors, especially among different adjustment and exposure models. A major problem is the reliable prediction of noise exposure levels among the different exposure models (*e.g.*, higher predictive quality of a specific model for air pollution will yield a more robust association of air pollution and outcome). Due to its nature, aircraft and railway noise is less associated with air pollution, whereas road traffic noise is usually highly connected to air pollution (especially in urbanized areas and city centers), leading to different confounding relationships between air pollution and different sources of traffic noise. Accordingly, railway and aircraft noise studies provide important prediction of environmental noise-related health effects that are less dependent on air pollution components than those obtained for road traffic noise. Although a considerable number of studies suggest that air pollution-induced health effects are independent of noise, the consideration of confounding and combined effects is important, especially with respect to traffic air pollution.

### G. Health effects of noise exposure in children

Children are a group that is extremely sensitive to noise exposure (254). This is simply because children are in a rapid growth and cognitive development phase and they have less developed defensive capabilities than adults to cope with environmental noise and can control noise less efficiently (234). As expected, noise exposure is causing an activation of the neurohormonal system in children (73). The Munich Airport study revealed that exposure to aircraft noise increases adrenaline and noradrenaline levels (71, 72). Hormone levels are elevated with increasing noise exposure time. No study has so far established an association of noise with cortisol excretion. There appears to be a minor positive association between aircraft noise and BP in children (184). Children are also as annoyed by traffic noise as adults. As shown in the Road traffic and Aircraft Noise exposure and children's Cognition and Health (RANCH) study, at 50 dB(A) there are 5.1% and at 60 dB(A) there are 12.1% annoyed by aircraft noise (257). Likewise, in the same study, a linear association was established for road traffic noise exposure with annoyance reactions (257).

There is a growing body of evidence that noise has detrimental effects on children's memory and reading outcomes. This has been shown for aircraft and also for road traffic noise (97). Further evidence for a noise-induced impairment of children's cognitive development was provided by intervention studies. In these studies, changes in noise exposure were clearly associated with changes in the cognitive performance (111). Also, a linear association of chronic aircraft noise exposure with impaired reading comprehension and reading memory was reported by the RANCH study (237). Taken together, the relationship between road traffic noise exposure and BP in Children is of minor nature, but its long-term effects could be more detrimental since children's health state often translates to the adolescent and adulthood health state. Obviously, environmental noise in children affects catecholamine excretion, annoyance, and confers negative effects on cognitive capabilities (*e.g.*, comprehension, long-term memory, and performance in standardized tests) (64).

What is the link between noise-induced cognitive impairment and oxidative stress? There are interesting new experimental data linking increased production of reactive oxygen species (ROS) by the enzyme NADPH oxidase (NOX2 subunit) in the brain with impairments in learning and memory (122). Thus, it is tempting to speculate that the cognitive impairments in children observed in response to noise may be at least, in part, mediated by increased NADPH oxidase-dependent superoxide production in the brain (122). Future studies have to address this topic, whether noise can cause an upregulation of the NADPH oxidase and an increase in superoxide production in the brain. Also, the sequence of events remains elusive, and whether cerebral oxidative stress precedes the adverse effects on the vasculature is not exactly known to this date. However, it is well established that noise-induced sleep deprivation (among other severe life stress factors) can cause oxidative stress in the brain, providing a link to neuronal disorders and memory impairment (8, 123, 206). Sleep deprivation *per se* can induce cerebral oxidative stress, inflammation, and increased angiotensin II signaling and cortisol release *via* activation of the hypothalamic–pituitary–adrenal (HPA) axis (206), all of which will also affect cardiovascular health (41) and providing a link between depression and CVD (173).

### H. Traffic noise mitigation strategies

The European Environment Agency has recently shown that the number of Europeans exposed to high levels of noise is on the rise. When comparing data between 2007 and 2012, they observed that there had been a general increase of people exposed to noise from airports; a slight increase of people exposed to noise from roads, and a slight decrease of people exposed to noise from railways. The rise in people exposed to road, rail, and airport noise is expected to increase worldwide during the next decade. The authorities may use different strategies to reduce the levels of road traffic noise.

Examples for effective measures causing road noise reduction are shown in Table 2. For aircraft and railway noise, introduction of night bans is a strategy for reducing exposure to noise during sleep, which is known to be an especially important exposure window in relation to health effects of noise. In addition, reduction of noise (aircraft noise) may also be achieved by implementing continuous descent arrivals (CDA), which require aircraft to approach on steeper descents with lower, less variable throttle settings. This CDA approach reduces noise, burns less fuel, and reduces emissions. Furthermore, installing sound-reducing windows is recommended in general, which will amount to ∼10 dB of reduction in indoor noise. Finally, more awareness with respect to road traffic noise when choosing tires for vehicles would result in a marked reduction in noise, as less noisy tires are available (5–10 dB difference) at similar costs to higher noise tires.

**Table 2.** Effective Mitigation Strategies Leading to Noise Reduction

*Change in noise, dB(A)*	*Perceived change*	*Examples on methods leading to reduction*
1	A very small change.	Reduce speed by 10 km/h, smoother traffic, shifting traffic from nighttime to daytime period, remove 25% of traffic
3	An audible, but small change.	Reduce speed by 20 km/h, using noise-reducing asphalt, remove 50% of traffic
5	A substantial and significant change.	Use noise barriers, remove 65% of traffic
10	Large change. Sounds like a halving of the sound.	Use high noise barriers, remove 90% of traffic

dB(A), A-weight decibels.

However, increasing population, urbanized areas, and socioeconomic factors require long-term strategies and policy directives. Even when the per-vehicle emissions of air pollutants and noise would be dramatically reduced, the yearly growing demand of human mobility and transportation of goods, a basic requirement for the growing economy world-wide, will compensate for these restrictive efforts. Long-term measures comprise urban planning (*e.g.*, minimum distances between sources and individuals, better sound-wall barriers, relocation of major trafficked roads and airports away from heavily crowded areas, prevention of industrial-residential areas, and better roads). Another primary strategy to reduce environmental risk by noise and air pollution includes better filter systems and noise isolation of buildings to prevent diffusion of outdoor air pollution and noise into indoor environments.

Also, scientific improvements in transportation technologies may help to reduce air pollution and noise in the future, although implementation may be hardly achievable in poor countries where cost is a primary consideration. In the meantime, acknowledgment of these environmental risk factors in clinical guidelines may put pressure on the government for more efficient reduction of air pollution and noise exposure limits. Future studies will have to address to what extent these mitigation measures will be able to protect from oxidative stress-induced vascular damage.

## III. Effects of Noise on Vascular Function and Oxidative Stress

Endothelial function has been demonstrated to be a biomarker reflecting increased oxidative stress in vascular tissue with prognostic meaning [for review, see Gori and Münzel (95) and Münzel et al. (163)]. There are only few interventional human studies investigating the adverse vascular effects of noise and our reports on the vascular consequences of aircraft noise in healthy subjects and patients with established coronary artery disease represent two examples (207, 208). Thus, the primary endpoint chosen in these translational vascular function studies was endothelial function as a parameter. To address the mechanisms underlying oxidative stress-induced endothelial dysfunction, we developed a novel animal model of aircraft noise exposure allowing us to identify the enzymes responsible for increased production of reactive oxygen and nitrogen species within the vasculature.

### A. Prognostic meaning of endothelial dysfunction and vascular oxidative stress

The endothelium-derived relaxing factor, which is now known since more than two decades as nitric oxide (^•^NO), and some of its reaction products (*e.g.*, S-nitrosothiols, iron/nitrosyl species or inorganic nitrite), confer potent antiatherosclerotic effects (95, 163). ^•^NO was formed by endothelial cells together with prostacyclin control platelet aggregation and vascular tone. ^•^NO also prevents adhesion of neutrophils to the endothelium, adhesion molecule expression, and smooth muscle cell proliferation (165). Therefore, ^•^NO deficiency contributes to atherosclerosis.

The half-life of ^•^NO, and accordingly its biological activity, is mainly influenced by ROS, especially superoxide (163). Superoxide interacts with ^•^NO in an almost diffusion-limited reaction yielding the intermediate peroxynitrite (ONOO^−^), a potent biological oxidant (163). The fast bimolecular reaction of ^•^NO with superoxide (rate constant: 5–10 × 10^9^ *M*^−1^·s^−1^) is approximately three to four times faster compared with the dismutation of superoxide by the superoxide dismutases (SODs) (28). Accordingly, peroxynitrite generation is a major sink of ^•^NO, but this process also activates ^•^NO, depending on the cellular superoxide levels. Higher formation rates of peroxynitrite are cytotoxic and induce oxidative modifications of proteins, lipids, and DNA (27). Peroxynitrite was also reported to adversely affect the activity and/or expression of prostacyclin synthase (282), endothelial NOS (133), and soluble guanylyl cyclase (sGC) (26). Hydrogen peroxide, formed by superoxide dismutation by SODs, and hypochlorous acid, produced by activated granulocytes, are also ROS but without free radical character. However, hydrogen peroxide and hypochlorous acid are also potent oxidants that can induce oxidative stress in vessels (28).

**FIG. 9.**
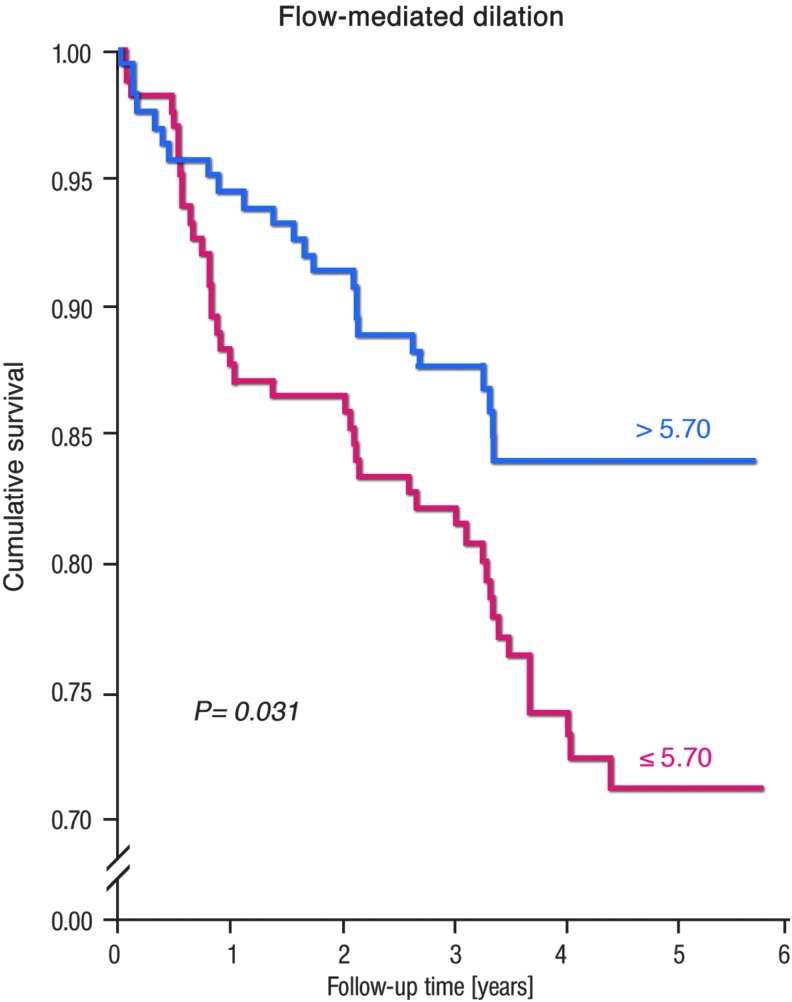
**Kaplan–Meier analysis of event-free survival refers to subgroups of patients categorized as being below and above the median values for mean FMD.** Lower FMD was associated with higher cerebro-cardiovascular event incidence. FMD, flow-mediated dilation. Reprinted from Ostad *et al.* (182). Copyright (2014), with permission from IOS Press. The publication is available at IOS Press through http://dx.doi.org/10.3233/CH-131720

During the last 17 years, an appreciable number of clinical trials have shown a close relationship between coronary and peripheral endothelial function and the risk of cardiovascular events. Thus, endothelial dysfunction has been observed in patients with arterial hypertension, subjects with normal BP but with a family history of hypertension, smokers, passive smokers, patients with dyslipidemia, aging patients, and those with diabetes mellitus [for review, see Flammer *et al.* (81)]. Using the methodology of assessing flow-mediated dilation (FMD) of the brachial artery by upper arm occlusion, we demonstrated that this approach has prognostic meaning in 325 patients with established coronary artery disease (Fig. 9) (182). Patients with established coronary artery disease and subsequent major adverse cardiovascular and cerebral events (MACCE), including cardiovascular death, myocardial infarction, and coronary revascularization by percutaneous coronary intervention or bypass surgery, hospitalization for unstable angina or decompensated heart failure, and nonhemorrhagic stroke, had a significantly lower FMD (+4.9%) compared with patients without MACCE (6.3% FMD).

However, a pharmacological proof of this concept is still missing since so far no interventional study was published that showed normalization of FMD (*e.g.*, by ^•^NO donors) improved the prognosis of patients, and even the prognostic value of FMD was questioned by several studies demonstrating that vascular function measurements without invasive interventions do not contribute much to the prognostic value of the European Society of Cardiology risk score (210) and was not independently correlated with cardiovascular events (240, 279). The pros and cons for FMD as a predictor of future cardiovascular events were discussed in detail previously (57).

**FIG. 10.**
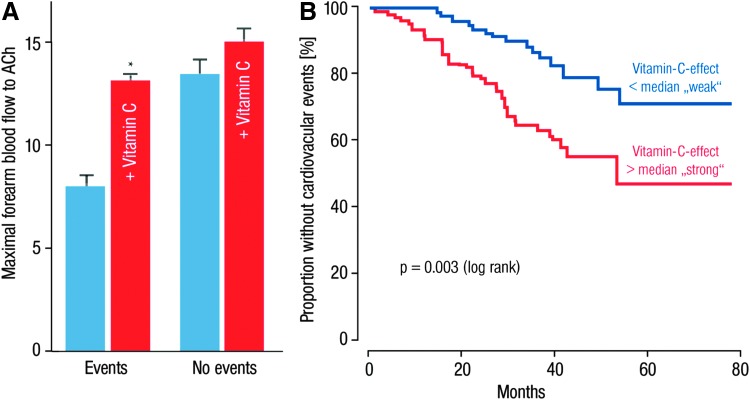
**Impact of oxidative stress on endothelial function and event-free survival of patients.** **(A)** Maximal ACh-induced vasodilation in patients with and without cardiovascular events during saline and vitamin C infusion. Vitamin C improved ACh-induced vasodilation significantly larger in patients with events compared with patients without events. **(B)** Kaplan–Meier analysis demonstrating cumulative proportion of patients without cardiovascular events during follow-up. Effect of vitamin C on ACh-induced vasodilation is divided into values below and above the median, *p* < 0.05 versus group without vitamin C (*blue bars*). Adapted from Heitzer *et al.* (106). With permission of Wolters Kluwer Health, Inc. Copyright © 2001, American Heart Association, Inc.

It is also of prognostic importance to what extent endothelial dysfunction is corrected by the acute administration of vitamin C, as this reflects the degree of oxidative stress within the vasculature (106). In patients with endothelial dysfunction and established coronary artery disease, Heitzer *et al.* established that endothelial dysfunction and higher burden of vascular oxidative stress represent useful predictors of future cardiovascular events (106). With these studies, the authors showed that endothelial dysfunction patients experiencing cardiovascular events, including cardiovascular mortality (*e.g.*, myocardial infarction, ischemic stroke, coronary angioplasty, and coronary or peripheral bypass operation), had greater benefit from vitamin C than patients without any cardiovascular event (Fig. 10A, B) (106).

Excessive ROS formation led to loss of progenitor repair capacity and contributes to the progression of atherosclerosis and adverse remodeling (193, 218), resulting in an imbalance between vascular damage and repair observed during the aging process (142, 146). However, it should be noted that ROS do not only affect the health of patients but also of healthy subjects and that ROS are not only detrimental but, at lower concentrations, confer important redox signaling, involving stress adaptation processes (112, 242) and other essential cellular functions such as cell migration and differentiation (65, 159). This may also explain why so far most interventional studies based on antioxidant therapy failed to improve the prognosis of the treated subjects (92, 209).

In addition, the effect of ascorbate infusion and age remained independent predictors of future cardiovascular events, even on adjustment for conventional risk factors that blunted endothelium-dependent vasodilation by acetylcholine, using a Cox proportional regression analysis (106). Thus, the degree of oxidative stress in the peripheral vasculature may go parallel with increased oxidative stress in the coronary arteries, thereby causing, for example, plaque rupture. Indeed, Sorescu *et al.* demonstrated that in atherosclerotic arteries, there was an intense area of superoxide in the plaque shoulder, which is rich in superoxide producing macrophages containing the gp91phox isoform of the NADPH oxidase (228).

**FIG. 11.**
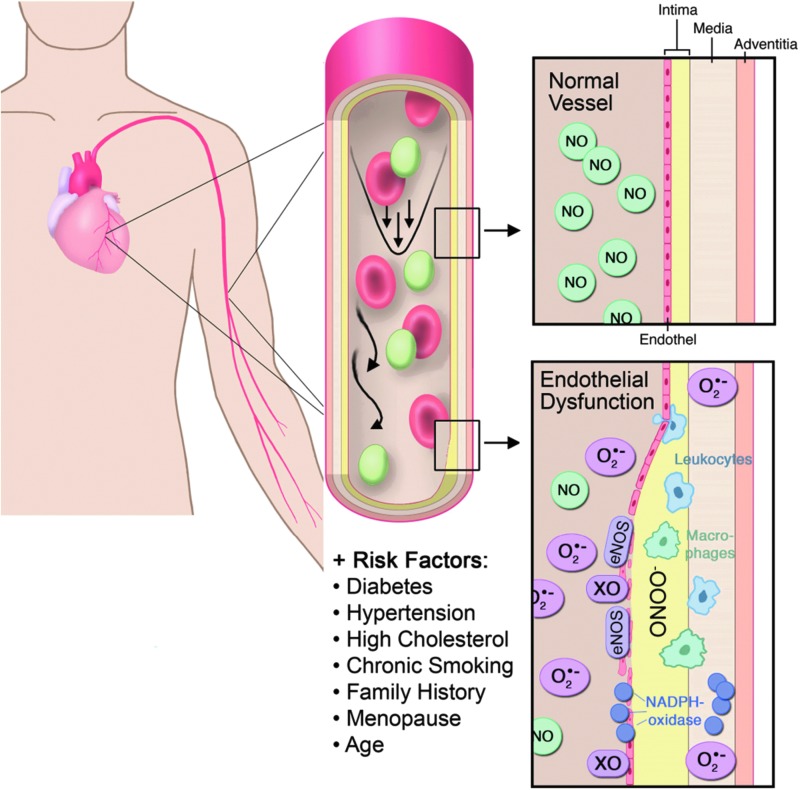
**Physiology and pathophysiology in the intact and damaged vasculature with direct effects of oxidative stress.** NO, nitric oxide; ONOO^−^, peroxynitrite; XO, xanthine oxidase. Modified from Daiber and Münzel (54a). With permission of the publisher. Copyright © 2006, Steinkopff Verlag Darmstadt.

The authors concluded that increased intracellular oxidative stress in human coronary atherosclerosis may be involved in the genesis and progression of human coronary atherosclerotic disease (228). Interestingly, the strong superoxide signal in the shoulder of the plaque and the intense staining of macrophages in the same area may indicate that superoxide produced by inflammatory cells may be at least, in part, responsible for plaque rupture and therefore for the clinical events such as an acute coronary syndrome (228). The most significant superoxide source within the vasculature causing endothelial dysfunction is vascular and phagocytic NADPH oxidase, uncoupled ^•^NO synthase, xanthine oxidase, and also the mitochondria (163). The direct effects of ROS on endothelial (vascular) function are shown in Figure 11.

Based on this evidence of the prognostic importance of the endothelium and the crucial role of oxidative stress in modulating endothelial function, we decided to choose endothelial dysfunction as our primary endpoint in endothelial function studies in healthy subjects (208) and patients with established coronary artery disease (207), as well as in a novel animal model of aircraft noise exposure (161).

### B. Noise and translational studies in humans

**FIG. 12.**
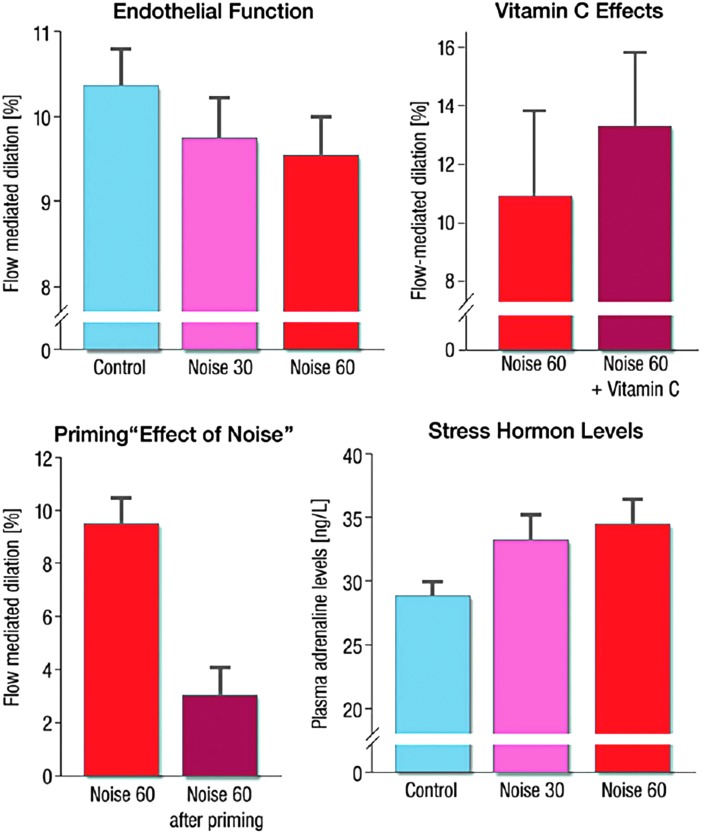
**Effects of simulated aircraft noise (noise 30 and 60 reflecting 30 or 60 playback aircraft noise events) on endothelial function (as measured by FMD) and (*lower right*) stress hormone levels of healthy volunteers.** The administration of the antioxidant vitamin C (*upper right*) was associated with improved endothelial function, demonstrating a role of oxidative stress. Adapted from Schmidt *et al.* (208) with permission of the publisher. Copyright © 2013, Oxford University Press.

In our recent field study, we found a correlation between the dose of nighttime aircraft noise exposure (in the subjects' bedrooms) and endothelial function as measured by FMD of the arteria brachialis (Fig. 12) (208).

The normalization of noise-induced endothelial dysfunction by acute administration of the antioxidant vitamin C pointed to an important role of oxidative stress in noise-triggered adverse vascular effects (Fig. 12). Vascular dysfunction was also associated with impaired sleeping quality and increased catecholamine production. There was also a “priming effect” of subjects who had previously exposed to noise, leading to a more pronounced adverse vascular effect of noise when exposed for the second time, suggesting sensitization of the vasculature to damage in response to repeated noise exposures. These findings provide a mechanistic explanation for a correlation between nighttime noise exposure and cardiovascular risk. More and more epidemiologic studies provide evidence that nighttime noise exposure has higher relevance for cardiovascular health than daytime noise exposure. The HYENA study observed no significant relationship for daytime noise and aircraft noise, but significantly increased BP with higher burden in nighttime noise (119).

In support of these findings, it has been shown that road traffic noise exposure is more detrimental in people who sleep with open windows or have a bedroom facing the road (20). A sustained nocturnal decrease in BP (so-called dipping) is required for resetting the cardiovascular system and for long-term cardiovascular health (205). Repeated nighttime autonomic arousals likely interfere with BP dipping and increase the risk for onset of hypertension in that part of the population with exposure to relevant levels of long-term environmental noise (102).

A recent study in Switzerland demonstrated a more detrimental effect of railway noise on BP during nighttime exposure (62). The Night Noise Guidelines for Europe were published by the WHO in 2009 and contain an important correlation of four noise exposure ranges to negative health outcomes, ranging from “no substantial biological effects” to “increased risk of cardiovascular disease” (WHO night noise guidelines). According to these guidelines, noise levels of L_Aeq,outside_ 55 dB(A) (see Table 1 for definition) are defined as a goal that can be achieved rapidly to prevent negative health effects of noise, in cases where the more favorable value of 40 dB(A) cannot be reached.

In summary, nighttime noise affects autonomic regulation [*e.g.*, by increased heart rate, sympathetic activation and/or parasympathetic withdrawal (33, 102, 145), and increased BP (42)] as well as vascular function (208). Of note, endothelial dysfunction and reduced heart rate variability were associated with the prognosis of patients with peripheral artery disease, arterial hypertension, and acute coronary syndrome or chronic stable coronary artery disease (36, 140, 165).

**FIG. 13.**
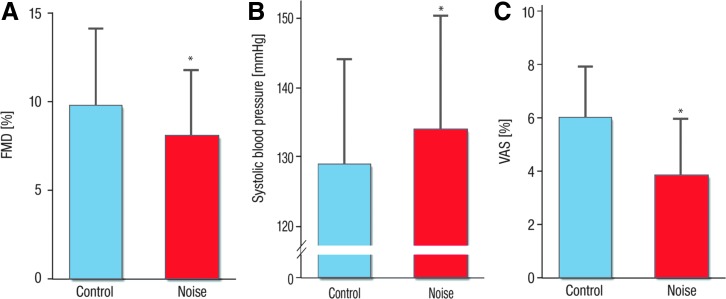
**Impact of noise exposure during the sleep phase on different cardiovascular and hemodynamic parameters.** Effects of nighttime aircraft noise on flow-dependent dilation **(A)**, on systolic blood pressure **(B)** and on sleep quality as expressed by the VAS **(A)** and data are mean ± SD in 60 patients with established coronary artery disease. Significance levels are *p = 0.001 for **(A)**, *p = 0.03 for **(B)**, and *p* < 0.001 for **(C)**, respectively, adjusted for gender, age, night sequence, PSQI, overall noise sensitivity (NoiSeQ), sleep-related noise sensitivity, attitude toward aircraft noise, and the results of the Morning Evening Questionnaire. PSQI, Pittsburgh sleep quality index; SD, standard deviation; VAS, visual analog scale. Adapted from Schmidt *et al.* (207) with permission of the publisher/authors. Copyright: © 2015, Schmidt *et al.* (open access).

In a similar study, even more pronounced cardiovascular effects of noise were observed in patients with established coronary artery disease and the study had to be terminated earlier (207). With our field studies, we demonstrated sleep disturbance and increased BP as well as endothelial dysfunction in response to noise exposure (207) (Fig. 13). Of note, nighttime noise impaired endothelial function independently of noise sensitivity and annoyance, probably by subliminal processes (207). Our observations provide a mechanistic basis for induction of endothelial dysfunction in patients with significant coronary artery disease by aircraft noise exposure, also providing an explanation for the epidemiological study results on more frequent arterial hypertension, chronic coronary artery disease, heart failure, stroke, and also cardiac arrhythmia in noisy, urbanized areas (166, 261).

### C. Noise and translational studies in animals

Noise in animal housing facilities is an inevitable environmental variable that can affect hearing, behavior, and physiology in mice (136). Noise levels in animal housing facilities can reach up to 80 dB(A), due to ventilation, and during human activity interference (*e.g.*, changing the animal cages), peak SPL of more than 100 dB(A) can be reached. These facts should be considered when planning noise exposure experiments. According to a review article by Turner *et al.*, the background noise level recorded from a representative animal housing unit (Southern Illinois University School of Medicine) was around 42 dB(A) with peak levels between 60 and 70 dB(A) (252). This review article also provides an excellent overview on noise susceptibility of different animal species and strains, their hearing frequencies, and common pathways of noise-induced adverse effects on health. Of great value are the examples for noise-induced cardiovascular, hormonal, biochemical, immunological, and other alterations (comparison of human and animal data). Most significant pathophysiological changes in response to noise include increased BP, vascular dysfunction, stress hormones, immunomodulation, slower wound healing, weight loss, and impaired fertility and reproduction.

#### 1. Direct pathway activation *via* ≥100 dB(A) noise exposure on the inner ear (hearing loss)

The contribution of noise and aging to hearing loss is distinct, yet interrelated (277). Noise-induced hearing loss (NIHL) is one of the leading causes of hearing loss worldwide. NIHL is usually characterized by an elevation in hearing threshold, and the area of damage is most pronounced one-half octave above the frequency of noise exposure. A significant body of evidence suggests that NIHL damage results from noise-induced free radical production after noise exposure (47, 80, 181, 219). Oxidative stress-dependent cochlear vascular dysfunction and inflammation were reported as a central player in NIHL (79, 246). Hearing loss could also be mimicked by administration of the redox cycler paraquat to chinchillas, which resulted in appreciable increases in cochlear superoxide formation and NIHL-typical threshold shifts (32).

In another chinchilla model of NIHL, increased cochlear NADPH oxidase and NF-κB activity was observed on noise exposure (190). Wistar rats exposed to noise with an SPL of 100 or 110 dB(A) (8–12 kHz) for 24 h displayed increased cochlear expression of NOX1 and DUOX2 but decreased expression of NOX3 (262). The latter finding seems to be at variance with a genome-wide association study identifying *Nox3* as a critical gene for susceptibility to NIHL (137). The contribution of mitochondrial ROS to NIHL is supported by aggravation of cochlear complications in mice with heterozygous deficiency in manganese superoxide dismutase (SOD2) (251), which also holds true for age-related hearing loss (125). Lack of other antioxidant enzymes such as Cu,Zn-SOD or glutathione peroxidase-1 also potentiates the complications of NIHL, further supporting a central role of oxidative stress in its pathogenesis (178, 179).

Age is regarded as an independent risk factor for acquired hearing loss, although the specific effects leading to the pathophysiological phenotype may not be easy to separate from those of noise exposure in the clinical setting (194). Despite the fact that the mechanisms underlying hearing loss in association with the aging process (presbycusis) are strongly influenced by the genetic susceptibility of a given subject, the pathophysiology of the aging process itself is highly interconnected with increased oxidative stress and low-grade inflammation (158, 239), reflecting major pathomechanisms in NIHL (194). Therefore, oxidative stress and inflammation may represent a link between noise- and age-related hearing loss and accordingly a unifying target in both complications. This concept is further supported by observations that high-intensity noise [105 dB(A)] induced more pronounced cochlear damage by aggravated impairment of cochlear blood flow in diabetic mice and also less efficient recovery in the hyperglycemic animals (87). This is an important observation since oxidative stress also plays a central role for the pathogenesis of diabetes (109, 176, 267).

Cochlear synaptic loss and hair cell death may play a role for noise- and age-related hearing impairment as observed on exposure to noise with an SPL of 100 dB(A) (8–16 kHz) for 2 h (132). When mice were exposed to that noise, there was a significant degree of synaptic loss and cochlear dysfunction starting at 16 weeks after exposure, which were not observed when mice were exposed to noise with an SPL of 91 dB(A) (8–16 kHz) for 2 h (77). The hearing loss even was evident 1 h after noise exposure, when noise with an SPL of 100 dB(A) at frequencies >22 kHz was applied for 2 h, indicating that higher frequencies are even more detrimental. The authors concluded that one single exposure to noise with SPL ≥100 dB(A) can accelerate cochlear aging. When guinea pigs were exposed to noise with an SPL of 106 dB(A) (4–8 kHz) for 2 h, ∼30% of the auditory nerve synapses on inner hair cells were lost, leading to cochlear neuropathy and partial hearing loss (77). Application of higher SPL of up to 120 dB(A) also results in structural damage in the mouse cochlea (183).

Reports on systemic effects of high-energy noise exposure were published for the prostaglandin E2-dependent hyperalgesia (increased inflammatory pain) of rats on exposure to a 105 dB tone with frequencies of 11–19 kHz (5 or 10 s per minute for 30 min per day) (124).

Among the practical studies of induction of hearing loss, magnetic resonance imaging-induced acoustic noise was investigated, and it was found that SPL of 100 dB(A) and more can be reached that may cause inner ear damage not only to humans (patients and personnel) but also to companion and experimental animals (135). When hearing loss was induced in guinea pigs by exposure to noise with an SPL of 106 dB(A) for 30 min, red blood cell velocity was reduced gradually over 2 and 3.5 h as measured by *in vivo* fluorescence microscopy (12). This indicates that cochlear microcirculation plays an important role for the development of hearing loss.

#### 2. Indirect, nonauditory vascular effects of ≤100 dB(A) noise exposure

There are only few experimental studies in animals on the mechanisms of noise-dependent effects on endothelial function and cardiovascular risk. When rats were exposed to noise for periods of 2 and 4 weeks (100 dB(A), 4 h/day, 6 days/week) endothelium-dependent vasodilation (measured by acetylcholine in the thoracic aorta) was impaired, the sensitivity to the vasoconstrictor serotonin, but not phenylephrine or potassium chloride was increased, and systolic BP was elevated by 31 mmHg (272). In a similar study, the same authors demonstrated an increase in systolic BP after 3 weeks (25 mmHg) and 4 weeks (37 mmHg) of noise exposure and pronounced endothelial dysfunction in isolated mesenteric arterial rings (273).

More recently, noise exposure of rats was reported to adversely affect the cardiovascular system by increased levels of circulating stress hormones (*e.g.*, corticosterone, adrenaline, noradrenaline, endothelin-1 [ET-1]) and negatively affected oxidative stress markers, such as increased malondialdehyde and decreased SOD, all of which point toward endothelial dysfunction [octave band noise: 80–100 dB(A), 8–16 kHz, 8 h/day for 20 days; 8 rats/group] (204). These elevated risk markers were associated with increased heart rate, mean arterial BP, and circulating nitrogen oxides, which could be a marker of ^•^NO coming from inducible NOS in inflammatory cells. Of note, these authors also compared different noise exposure protocols, and the most pronounced adverse effects were observed in the chronic exposure group (8 h/day for 20 days), whereas the single exposure for 12 h induced moderate impairments that were comparable with those observed on chronic intermittent noise exposure (8 h/day for a total of 20 days with 3 days of exposure followed by 2 days of cessation) (204). Also, oxidative DNA damage (detected by the comet assay) was observed in the rat heart and adrenal gland on noise exposure ≥100 dB(A) (86, 139). Cardiovascular therapy using the cholesterol lowering substance rosuvastatin effectively prevented these adverse effects (70).

Likewise, exposure of rats to white noise (90 dB, 15 min daily for 3 weeks) caused adverse effects on the morphology of the intestinal mucosa with separated or even detached mucosal epithelial cells and evidence of edema (24). Recovery of rats for another 3 weeks improved epithelial integrity and partially the function of mucosal cells. Using a similar protocol, the same authors showed that noise exposure induces mesenteric microvascular structural damage with increased number of leaks that was significantly reduced by anti-inflammatory and antioxidant cotreatment (23). White noise exposure of 100 dB with frequencies of 0.4–6.3 kHz (4 h per day for 30 days) caused a transient increase in blood glucose, markers of inflammation, triglycerides, and changes in the microbiome that returned to normal at 14 days after cessation of noise exposure (53).

In 1981, a study reported on increases of mean BP by 30 mmHg in response to noise exposure [mean SPL of 85 dB(A), six episodes per day for 9 months, 97 dB(A) peak level] in two chronically exposed monkeys without significant effects on the auditory system (185). In another study from the year 1992, rats were exposed to noise with an SPL of 85 dB(A) for 12 h/day for 8 weeks and subsequently with an SPL of 95 dB(A) for 16 h/day for 4 weeks, and an increase of systolic and diastolic BP by 16 mmHg was observed along with magnesium deficiency and reduced lumen sizes of microvessels (7). Exposure of guinea pigs to noise [70 or 90 dB(A)] resulted in gradual loss of vascular endothelial growth factor (VEGF) in the cochlea. VEGF is a proangiogenic factor that regulates vascular permeability and also displays neuroprotective properties (217). Exposure of rats to moderate noise with an SPL of 70 or 85 dB(A) (8–16 kHz; 6 h/day for 3 months) led to neuroendocrine modulation with increased corticosterone levels and increased lipid peroxidation being more pronounced in the 85 dB(A) group, whereas upregulation of antioxidant enzyme catalase and SOD was similar in both exposure groups (89). In addition, the authors reported on morphological changes in the heart [*e.g.*, inflamed area of pericardium and dilated veins on 70 dB(A) exposure and more dilated veins in the periphery of pericardium on 85 dB(A) exposure] and analog changes in tissues of the thyroid and adrenal gland.

#### 3. Indirect, nonauditory pathway activation with noise exposure ≤85 dB(A)

**FIG. 14.**
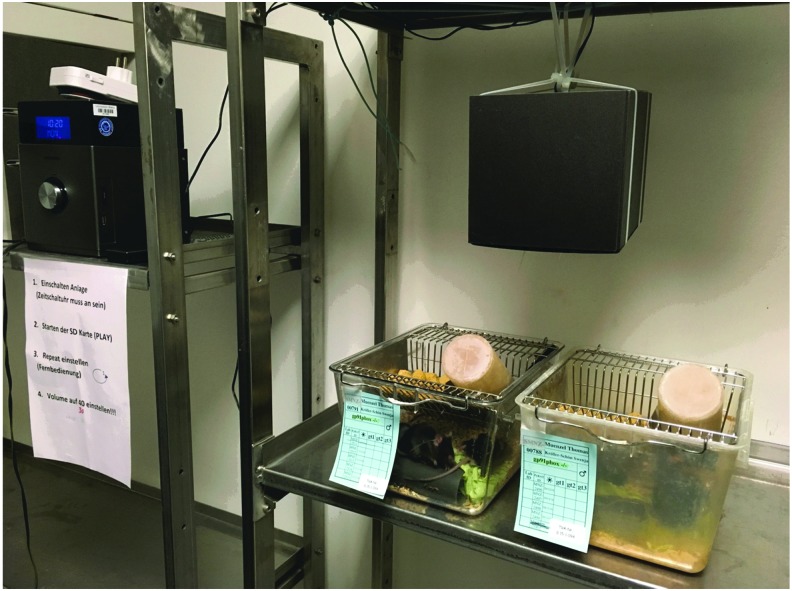
**Setup of the noise exposure system used for the mouse studies.**

Our recently established protocol of noise exposure to study the effects of aircraft noise on vascular function consisted of repetitive playback of a 2-h-long noise pattern (69 aircraft noise events with a duration of 43 s) and a maximum SPL of 83 dB(A). The noise events were separated by irregularly distributed silent periods to prevent early adaptation. Downward facing loudspeakers were used for the playback of the noise pattern and were mounted ∼30 cm above the plastic mouse cages (Fig. 14).

Playback of the noise pattern mp3 files was carried out using a Grundig MS 540 compact sound system with a total output of 65 W. Loudness and corresponding SPL were calibrated with a Class II sound level meter (Casella CEL-246) within one of the cages at initial setup. Actual SPLs during exposure were continuously recorded during the study period using the same device placed between cages with an upward facing microphone. The average SPL (equivalent to dB L_eq_) was 71.6 dB(A) with a usual intermediate background noise level of 50–55 dB(A) in the animal facility depending on the specific room. For investigation of the importance of specific noise patterns and characteristics, “white noise” was used as a control sound exposure with exactly the same average SPL and compared to the impact of continuous aircraft noise exposure for 1, 2, and 4 days on vascular function. “White noise” consists of a random noise with constant spectral density within the range of human hearing with frequencies of 20 Hz to 20 kHz.

**FIG. 15.**
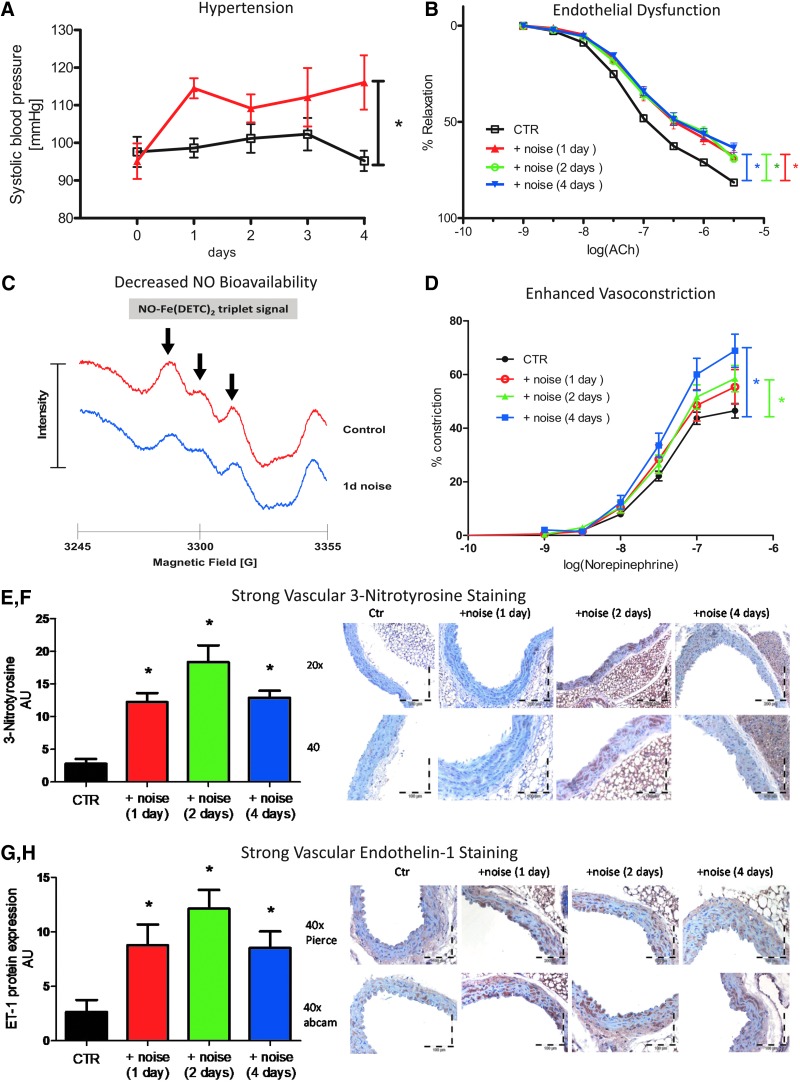
**Selected data of our recently published noise exposure mouse study.** Exposure to aircraft noise led to elevated systolic blood pressure (*red symbols*) **(A)**, impaired endothelial function **(B)**, reduced vascular NO levels [measured with EPR as described (128)] **(C)**, and enhanced sensitivity to vasoconstrictors **(D)**. Exposure to aircraft noise led to increased staining of vascular 3-nitrotyrosine-positive proteins **(E, F)** and vascular autocrine endothelin-1 production **(G, H)**. The staining reflects representative immunohistochemical images. *Brown color* indicates 3-nitrotyrosine-positive proteins or endothelin-1 expression and localization. EPR, electron paramagnetic resonance spectroscopy; ET-1, endothelin-1. Modified from Münzel *et al.* (161) with permission of the publisher/authors. Copyright © 2017, Münzel *et al.* (open access). Published by Oxford University Press on behalf of the European Society of Cardiology.

Already after 24 h of continuous exposure to aircraft noise, we observed increased stress hormone levels for catecholamines Ang II and ET-1 (161). Also, oxidative stress markers such as 3-nitrotyrosine- and malondialdehyde-positive proteins and endothelial (vascular) dysfunction, as well as BP, were increased on noise over the entire exposure period relative to pre-exposure levels (Fig. 15). The increase in the NOX2 protein (phagocyte NADPH oxidase) content in the aorta of mice exposed to aircraft noise pointed toward increased infiltration of inflammatory cells into the vasculature as shown by FACS analyses (161). A major mechanism of the development of arterial hypertension consists of vascular infiltration of inflammatory cells leading to endothelial dysfunction and oxidative stress, previously demonstrated by genetic/pharmacological deletion of LysM-positive cells (265) and recently reviewed in detail (266).

**FIG. 16.**
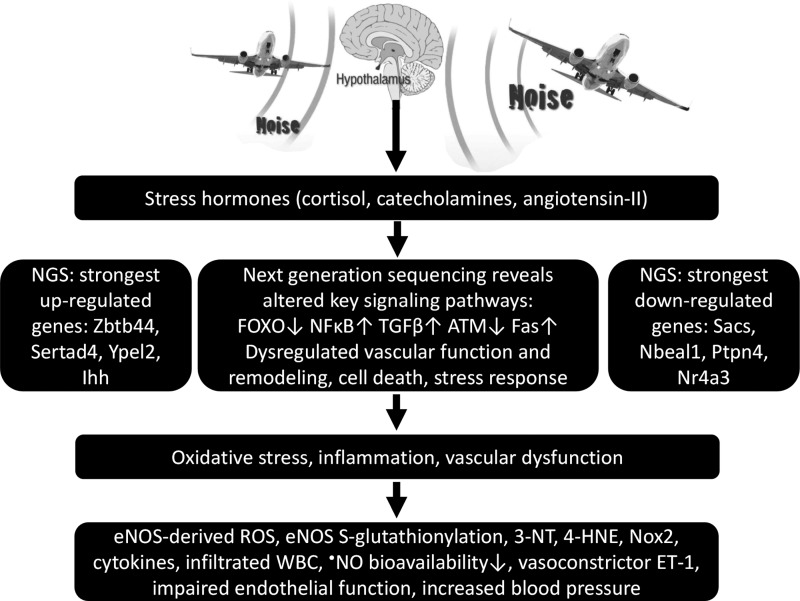
**Postulated mechanisms of noise-induced (cardio)vascular damage are based on the vascular functional and observational parameters as well as results from next-generation sequencing of our recent mouse study on the effects of aircraft noise exposure on the cardiovascular system.** eNOS, endothelial nitric oxide synthase; Foxo, Forkhead-box-protein; Ihh, Indian hedgehog; ^•^NO, nitric oxide; Nox2, NADPH oxidase isoform 2, ROS, reactive oxygen species, Ypel2, Yipee-like 2.

Endothelial nitric oxide synthase (eNOS) uncoupling was revealed in the aorta and hearts from animals exposed to aircraft noise (161). The presence of uncoupled eNOS was supported by increased levels of endothelial ROS formation, which was inhibited by the eNOS inhibitor L-N^G^-nitro-arginine [L-NAME; using the dihydroethidium cryostaining method (55)]. As a surrogate parameter for the uncoupling reaction, eNOS S-glutathionylation was found elevated in the heart and aorta on noise exposure (summarized in Fig. 16) (44).

GTP-cyclohydrolase-1 and dihydrofolate reductase are responsible for the synthesis and recycling of tetrahydrobiopterin (BH4), which is oxidized by high intracellular oxidative stress to the ^•^BH3 radical, thereby causing functional depletion of the eNOS cofactor (29, 134, 212). The here observed increased expression of these enzymes in the noise-exposed groups points toward compensatory upregulation of these enzymes to overcome eNOS uncoupling and endothelial dysfunction. Accordingly, the ^•^NO bioavailability was decreased in aorta of noise-exposed mice (measured by electron paramagnetic resonance spectroscopy). The increased sensitivity to vasoconstrictors, such as norepinephrine, and the elevated vascular ET-1 immunohistochemical staining further supported the observed endothelial dysfunction in response to noise exposure (Fig. 15).

Of note, exposure to “white noise” at similar mean SPL did not induce these adverse vascular effects in response to aircraft noise exposure, clearly indicating that not only the loudness (quantity) of a specific noise but also its characteristics (quality such as frequencies, pattern) determine its harmful effects (161).

#### 4. Effects of aircraft noise on vascular gene regulation as established by next-generation sequencing

We were also able to identify significant alterations in gene expression profiles by using next-generation sequencing (NGS) (summarized in Fig. 16) (161). Pathway analysis by gene ontology annotation from NGS data revealed major changes in the vascular smooth muscle cell (VSMC) contraction pathway and TGFβ- and Smad signaling. Significant changes were detected in the NFκB-related pathway, adrenergic signal transduction, focal adhesion, cell cycle control, apoptosis, and kinase-mediated growth and proliferation signaling centered around the Forkhead-box-protein (Foxo) transcription factors herein (stress response and antioxidant defense) (161). The four strongest upregulated genes compared to controls were *Zbtb44*, *Setad4*, *Ypel2*, and *Ihh* and, similarly, a number of transcripts *Sacs*, *Nbeal1*, *PTPN4*, and *NR4A3* were significantly reduced by noise (summarized in Fig. 16). These genes are quite novel in the context of cardiovascular physiology and are generally poorly understood. Therefore, their exact role in noise-induced pathophysiology warrants further investigation. Changes were also observed in expression of genes being involved in the regulation of the circadian rhythm, insulin, and calcineurin signaling pathways, although the relationship of these pathways to noise-induced vascular damage was not addressed in detail so far.

*Zbtb44* codes for a highly conserved zinc finger domain DNA binding protein. It is involved in stem cell growth and responds to steroid and adrenergic stimulation (35, 271). The specific function in the cardiovascular vessels has not been determined. SERTAD4 is highly expressed in adult murine fibrous tissues. SERTAD4 resides predominantly in the nucleus throughout cell cycle progression and shows interaction with PP2A and PI3K (93, 186), which are crucial regulators in the pathways discussed here. Yipee-like 2 (YPEL2) is localized in the nucleus and contains several metal binding sites, it is known to interact with phosphatases and influences calcium signaling in cellular repolarization (11). Indian hedgehog (IHH) is involved in signaling and has been found among other pathways to participate in cartilage degeneration as well as in TGFβ-driven chondrogenesis and ossification (99, 263). Sacsin is highly expressed in the central nervous tissue and also shows abundant expression in fibrous tissue. It has chaperone-like function and is regulated by TGFβ in epithelial–mesenchymal transition (241). NBEAL1 is known to be strongly expressed in the mouse aorta; its lysosomal import sequence may implicate a role in autophagy and hypoxia (45).

PTPN4 belongs to a superfamily of protein phosphatases that is associated with cytoskeletal proteins. It has a role in cell growth and motility in various tissues and cell lines (281). Downregulation of the nuclear orphan receptor NR4A3/NOR1 is known to contribute to the regulation of matrix metalloprotease in vascular tissue and to the activation of VSMCs (197, 198). Conservative promoter database analysis of transcription factor binding sites of the corresponding gene products showed many binding sites with redox-active and cysteine-rich transcription factors (NF-κB, FOXO, and zinc-finger proteins) in the promoter regions of the eight most regulated genes (161). This might insinuate that noise-generated nitro-oxidative stress may directly influence transcription levels. Although these genes and their gene products are not well known in the cardiovascular context, through their interaction partners, they strongly contribute to the pathways discussed here.

ROS play a key role in linking the different pathways in the present NGS analysis (summarized in Fig. 16) (161). The IGF-1/insulin/PI3K/Akt pathway, for instance, can be activated *via* redox-sensitive mechanisms (10). Subsequently, Akt regulates the activity of FOXO transcription factors by phosphorylation, which has a transcriptional factor binding site on the *Nr4a3*, *Sod1*, and *glutathione peroxidase 1* gene promoter explaining at least, in part, the observed changes in the transcription level of the NGS experiment (253). Oxidative stress also directly regulates transcription factors FOXO and NF-κB through reversible oxidation and reduction of cysteine residues (147), all of which may affect other transcription factors such as cysteine-rich zinc-finger proteins such as ZBTB44. The changes in FOXO factor activity and other transcription factors may modulate cellular resistance capacity to oxidative stress by regulating key detoxification enzymes (*e.g.*, SOD2 and catalase) (147). By a similar mechanism, TGF-β1 contributes to oxidative stress *via* decreasing the expression of antioxidant enzymes (*e.g.*, SOD, catalase, glutathione peroxidase) (196).

These events may result in increased vascular ROS production and reduced vascular NO-bioavailability in favor of the formation of the NO/superoxide reaction product peroxynitrite, leading to decreased cyclic guanosine monophosphate (cGMP) concentrations and sGC sensitivity along with an inhibition of the activity of the cGMP-dependent protein kinase I (cGK-I), which is able to induce major structural and transcriptional changes in smooth muscle cells. cGK-I may interact with the TGFβ/Smad pathway, MAPK-signaling, as well as PI3K and FOXO pathways (76, 94, 144), which show relevant changes in the NGS transcription data. Some of the pathways modulated by nitro-oxidative stress represent pathological mechanisms potentially mediating the detrimental effects of noise, while others are likely to reflect compensatory mechanisms in response to noise-induced injury.

Despite the presented evidence for potential redox regulation of vascular gene expression by noise exposure in multiple signaling pathways, we should keep in mind that the presented NGS data do not reflect posttranslational modifications of proteins (including kinases, phosphatases, and transcription factors), which undergo substantial changes in an oxidative stress milieu, largely affecting enzymatic function *via* sulfoxidation, S-nitros(yl)ation, S-glutathionylation, and phosphorylation. Accordingly, the overall impact of redox regulation on these multiple signaling pathways may be even more pronounced at the proteomic or metabolic level than expected by the established changes at the genomic level. These observations are also in good accordance with a recent overview on the impact of environmental stressors such as noise on redox-regulated epigenetic pathways published within this Forum (157).

#### 5. Summary and conclusions of aircraft noise exposure in mice

Taken together, we propose the following pathophysiological sequence of events causing vascular damage in response to aircraft noise exposure (Fig. 16). Aircraft noise exposure leads to an overactivation of the sympathetic system, resulting in elevated levels of noradrenalin (NA), adrenalin (A), angiotensin II (Ang II), and subsequently cortisol. Angiotensin II, in turn, activates endothelial NADPH oxidase causing oxidative stress, which may induce direct scavenging of ^•^NO and eNOS uncoupling through oxidation of BH4 and eNOS S-glutathionylation. ROS play a key role in linking different pathways, including PI3K/Akt signaling, the FOXO transcription factors, TGF-β1 and NF-κB signaling, as well as the ET-1 system, increasing the circulating levels of IL-6, and the expression of vascular adhesion molecules.

Superoxide and ^•^NO produced by infiltrating immune cells (neutrophils, natural killer cells, and monocytes/macrophages) promote the formation of 3-nitrotyrosine-, malondialdehyde-, and 4-hydroxynonenal-positive proteins and inflict additional cellular oxidative damage. The uncoupling of eNOS not only reduces NO production but also potentiates the pre-existing oxidative stress. Endothelial NO production is further reduced by glucocorticoids such as cortisol, leading to impaired vasodilation and increased BP. The overproduction of noradrenalin, adrenalin, and ET-1 enhances contraction, which is further potentiated by glucocorticoids. All of these vascular alterations support the development of metabolic disorders as envisaged by increased blood glucose levels. ET-1 expression levels were also reported to contribute to the phenotype of Alport (Col4a3^−/−^) mice that display a particular susceptibility to toxic noise and represent a model of glomerular disease associated with hearing loss (151).

## IV. Adverse Effects of Simultaneous Noise and Air Pollution Exposure

### A. Adverse effects of noise and air pollution exposure share similar pathophysiological pathways

**FIG. 17.**
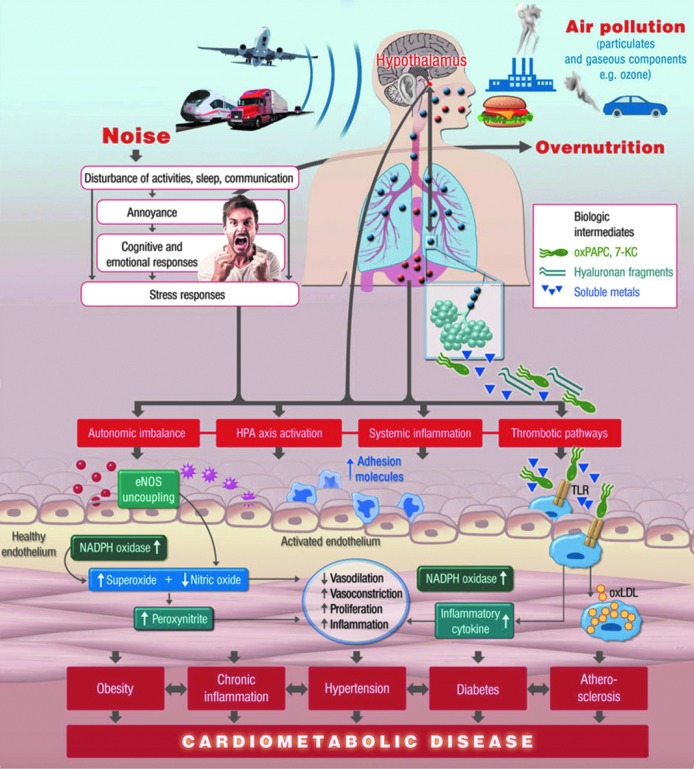
**Proposed pathophysiological mechanisms of cardiovascular disease induced by environmental air pollution and noise.** 7-KC, 7-ketocholesterol; ox-PAPC, oxidatively modified 1-palmitoyl-2-arachidonoyl-sn-phosphatidylcholine. Adapted from Münzel *et al.* (166) with permission of the publisher. Copyright © 2016, Oxford University Press.

Recent data support the idea that air pollution and noise, similar to traditional risk factors, contribute to vascular (endothelial) dysfunction, hypertension, atherosclerosis, and other cardiovascular events such as myocardial infarction, stroke, and congestive heart failure. In general, four pathways have been introduced to better explain the combined effects of air pollution and noise (Fig. 17): (i) a dysregulated autonomic nervous system and/or an activated sympathetic system; (ii) generation of mediators of inflammation, modified lipids, or phospholipids, and recruitment of different immune cell populations; (iii) oxidative impairment of endothelial dysfunction; and (iv) higher activity of prothrombotic signaling pathways (25, 66, 96, 207, 208). These adverse pathways may overlap and act synergistically, and may be activated at different points in time to noise or air pollution exposures, and have probably varying impact on cardiovascular events. The immediate effects observed within seconds/minutes/hours after noise or particulate matter (PM) exposures are most probably conferred *via* alterations in autonomic tone and/or activation of the sympathetic system, impairment of endothelial function (161), increased levels of procoagulant proteins leading to higher activity of procoagulant pathways, and more frequent thrombotic events [for review, see Münzel *et al.* (167) and Newby *et al.* (174)] (Fig. 17).

Acute effects of air pollution and noise pollution are more likely in patients with higher susceptibility (*e.g.*, with pre-existing cardiovascular complications such as “vulnerable plaque,” “vulnerable myocardium” (arrhythmias), or “vulnerable circulation”). Repeated exposures to environmental stressors mainly depend on the accumulation (duration of exposure), and accordingly, effects of noise and air pollution can be additive or synergistic. On chronic exposure also habituation/adaptation may occur, implying that most severe noise-induced physiologic effects are more likely within the first days of exposure. Likewise, the effects of noise observed in the field (where noise exposure of subjects for many years is frequently encountered) are usually less pronounced than those measured in laboratory conditions, where often exposures to unusual noise events are applied (25, 162, 187). Although a weaker response to air pollution on repeated exposure challenges would be in line with biological adaption processes, this was never shown in detail in a controlled experimental setting. Under specific circumstances, however, contrary observations could be made meaning that the second exposure may lead to more pronounced effects than the initial exposure, a physiological and medical phenomenon that is well known as “priming” (208).

### B. Gaps in current knowledge concerning noise and air pollution

**FIG. 18.**
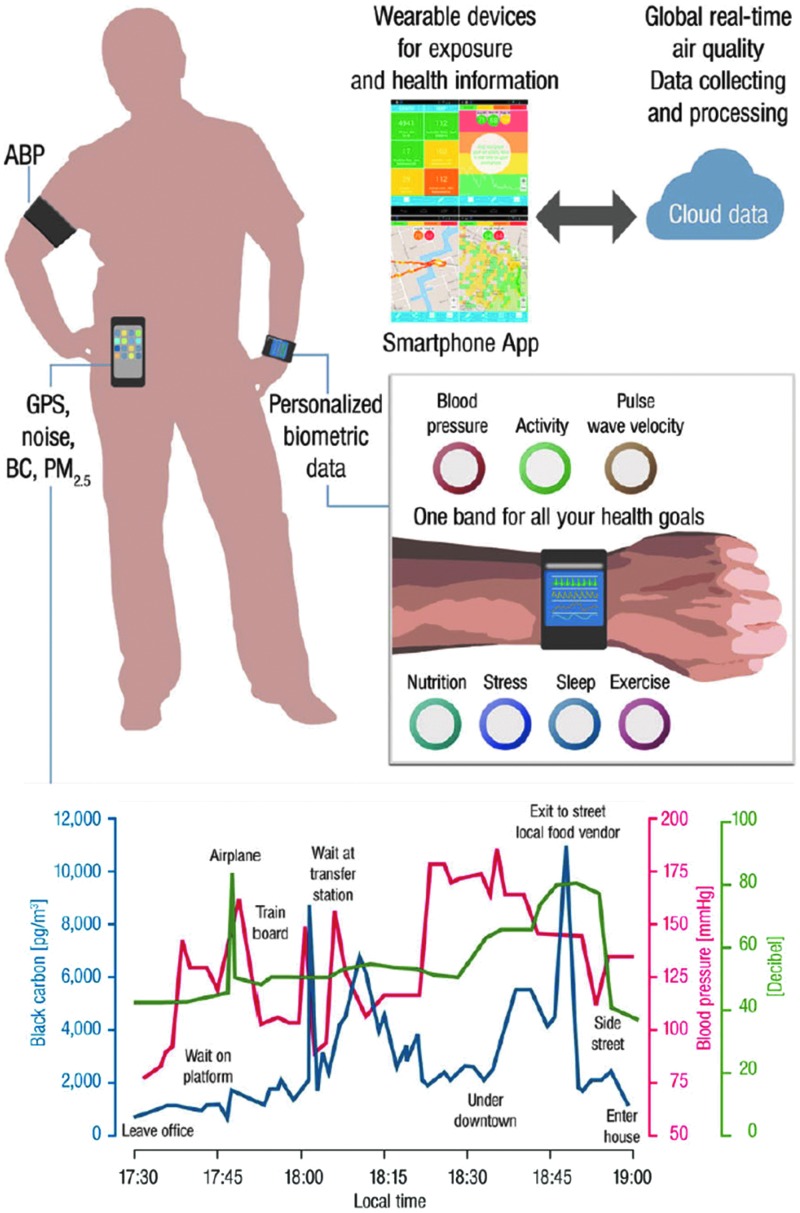
**Hypothetical framework of investigations that combine technological innovation in biometric data with personalized exposure information in real time to study interactive effects of environmental risk factors on cardiovascular endpoints.** ABP, ambulant blood pressure monitoring; BC, black carbon; PM, particulate matter. Adapted from Münzel *et al.* (167) with permission of the publisher. Copyright © 2016, Oxford University Press.

Only few studies investigate the additive or synergistic effects of simultaneous noise and air pollution exposure in humans (for animal research, we know not a single study addressing this issue). Studies in animals could answer important questions on the mechanisms of detrimental health effects by combined noise and air pollution exposures, providing the directions for future human studies. The questions comprise the extent and kinetics of response to coexposure, interactive effects of both factors on surrogate parameters (*e.g.*, BP and metabolic risk), reversibility of effects, impact of low-grade background noise on air pollution exposure effects and vice versa, modulation of the circadian rhythm, and improvement by preventive measures and lifestyle changes (*e.g.*, diet, stress, and exercise). Finally, new medical devices that allow to record health parameters as well as exposure levels to environmental pollutants may provide an important opportunity for research and understanding of the interactions of environmental with nonenvironmental risk factors (Fig. 18).

## V. Other Environmental Risk Factors, Oxidative Stress, and Cardiovascular Disease

Exposure to environmental pollutants is a major but significantly underestimated risk factor that contributes to the onset and progression of CVD (52). The cardiovascular system is susceptible to damage by various environmental risk factors such as air pollution (*e.g.*, PM or reactive nitrogen/sulfur oxides) and heavy metals (*e.g.*, arsenic, cadmium, or lead). These environmental risk factors may, similar to the traditional risk factors (*e.g.*, smoking, hypertension, and diabetes mellitus), increase the global burden of disease and mortality, also by adverse effects on regulatory pathways of vascular tone (leading to arterial hypertension), metabolism (leading to hyperlipidemia, obesity, and diabetes), and atherosclerosis. There is clear evidence that people living in highly polluted areas are at higher cardiovascular risk but more recently, there is also growing body of evidence that chronic exposure to these environmental risk factors, even at concentrations below the legal thresholds, leads to adverse effects on cardiovascular health.

Since most of these environmental pollutants are omnipresent, even their minor impact on cardiovascular risk has dramatic effects on public health (not only on mortality but also life years spent with severe illness and disability). Accordingly, as outlined above for the prevention of noise-induced cardiovascular damage, mechanistic and epidemiological studies have been conducted or will address in the future how mitigation strategies can help to decrease the adverse health effects of air pollution and heavy metals. The list of environmental toxic compounds affecting cardiovascular health could be extended at will [*e.g.*, pesticide-driven oxidative stress (63, 259) and CVDs (129, 215)], but in the present review with the focus on environmental noise effects on cardiovascular function, we only want to mention air pollution representing another major environmental cardiovascular risk factor (166, 167) that is mostly associated with noise exposure (*e.g.*, traffic generates noise and air pollution and these stressors cannot be easily separated). Other environmental stressors are only briefly mentioned, mostly since they are discussed in detail in the reviews included in this Forum.

### A. Air pollution (PM/carbon black)

In contrast to the research on noise-induced cardiovascular risk, the cardiovascular effects of air pollution are well characterized demonstrating vascular (endothelial) dysfunction, vascular inflammation and, in the longer run, the development of atherosclerosis (192). In addition, exposure to PM increases oxidative stress within the vasculature and increases the sensitivity to vasoconstrictors (280). Recent publications based on epidemiological and experimental studies suggest additive damage of noise on air pollution-induced CVD (166, 234), although no study has ever investigated the functional and molecular mechanisms of the interaction of both environmental stressors for oxidative stress and the vasculature itself. In addition, the Heinz Nixdorf Recall study showed that environmental stress, for example, air pollution and traffic noise, is independently associated with increased aortic calcifications (121), another predictor of future cardiovascular events, which warrants detailed basic science studies to gain more mechanistic insight in the pathophysiology of these potential additive adverse effects at the vascular level. In a small cohort trial, 18 healthy individuals were subjected to diesel exhaust at 276 g/m^3^ from a passenger car or filtered air for 3 h, with coexposure to traffic noise at 48 or 75 dB(A) (107).

Exposure to diesel exhaust had no effects on genotoxicity, oxidative stress, or inflammation in white blood cells isolated from the subjects, whereas exposure to noise caused oxidative DNA damage. In another study, 18 highway maintenance workers were monitored with respect to their exposure to PM and traffic noise, significantly associated with C-reactive protein, serum amyloid A, increased heart rate variability, or systolic and diastolic BP (152). The impact of PM and diesel exhaust on oxidative stress pathways and inflammation was reviewed in full detail in two articles within this Forum (191, 270).

As outlined in a recent review article (52), around 7% of nonfatal myocardial infarctions (171) and 18% of sudden cardiac deaths (104) are potentially triggered by exposure to road traffic-dependent air pollution, representing comparable numbers to those published for the contribution of the major traditional and modifiable risk factors, from smoking, poor diet, or obesity to cardiovascular morbidity and mortality (104). Despite the relatively low contribution of air pollution to the individual risk of a single human being, the cumulative cardiovascular risk conferred by chronic exposure of a large part of the population to ubiquitous air pollution (the cumulative global disease burden) even ranks above physical exertion, coffee, and alcohol in a comparative risk assessment of the major triggers of myocardial infarction (171). Only diet, high BP, and smoking represent more important cardiovascular risk factors for life years with severe illness and disability than air pollution (278).

Strikingly, the short-term pollution-control activities (mainly reduction in traffic and industrial air pollution) applied during the 2008 Beijing Olympic Games led to a 13–60% reduction in the concentrations of air pollutants, which were perfectly mimicked by similar effects on biomarkers of inflammation, oxidative stress, and thrombosis in healthy adults (110, 126, 195, 203). Unfortunately, the beneficial effects quickly returned back to normal when the restrictions for air pollution were invalidated after the Olympic Games.

An important feature of the ENNAH Network (see also the Introduction section) was the involvement of researchers mainly working on air pollution allowing joint considerations of the effects of both environmental risk factors, transportation noise and air pollution on public health (3). The EU made efforts in supporting cohort studies on the effects of air pollution on public health by substantial funds. ENNAH provided an excellent platform for exploitation of existing European cohort data that provide reliable information on air pollution and enriching these cohort data on air pollution with noise components from existing noise maps. These joint studies may have direct impact on transportation and environmental guidelines with respect to future policies in air pollution and/or noise thresholds and the development of new mitigation strategies. Therefore, the ENNAH Network represents one of the first large initiatives to plan future research on the synergistic effects of air pollution and noise exposure on public health.

### B. Other environmental stressors

Chronic mental stress in turn has been demonstrated to generate its own cardiovascular risk factors such as increased BP and dyslipidemia, increased blood viscosity and blood glucose, and activation of blood clotting factors (13). Findings from a large pan-European epidemiological study in 124,808 diabetes-free subjects indicate that job strain is a risk factor for type 2 diabetes in men and women independent of other lifestyle factors such as obesity and physical inactivity (175). A meta-analysis of nine case/control and cohort studies of good methodological quality showed positive associations between hypertension and job strain (22). The impact of mental stress on oxidative stress pathways and inflammation was reviewed in full detail by Siegrist and Sies (221) and in two articles within this Forum (155, 274).

Environmental exposure to electromagnetic radiation (EMR) has been increasing with higher demand for advanced communication infrastructure (*e.g.*, Wi-Fi) and devices (smart phones). The effects of EMR on oxidative stress, inflammation, and reproduction were previously reviewed (172). However, a recent critical review led to the conclusion that standardized protocols are required to obtain reliable data leading to better understanding of the underlying mechanisms (90).

As outlined in a recent review article (52), there is substantial evidence from epidemiological and experimental data suggesting that heavy metals [*e.g.*, cadmium (248), a systematic review of 31 studies and lead, a systematic review of 12 studies (170)] and metalloids [*e.g.*, arsenic, a systematic review of 12 studies (160)] can trigger CVDs. A modulation of BP, lipid metabolism, atherogenesis, and endothelial function was observed in response to these compounds (188). In the plasma, serum, and atherosclerotic lesions of mice treated with arsenic, increased levels of proinflammatory chemokines, cytokines, and markers of oxidative stress were observed (232).

Likewise, cadmium leads to vascular damage, endothelial dysfunction, and atherosclerosis by oxidative mechanisms (*e.g.*, by replacement of iron and copper in sulfur complexes, promoting Fenton reactions) (153, 154), interference with antioxidant responses (*e.g.*, by disruption of zinc/sulfur complexes) (54), and inhibition of ^•^NO-mediated vasodilation (6). Of note, lead inhibits the ^•^NO/soluble guanylate cyclase signaling pathway, stimulates the renin–angiotensin–aldosterone system and the sympathetic nervous system, and activates protein kinase C activity, all of which resemble strikingly the adverse effects of noise exposure on the cardiovascular system (258). The impact of environmental chemicals such as heavy metals on oxidative stress pathways and epigenetic gene regulation was reviewed in full detail in an article within this Forum (157).

## VI. Summary and Future Perspectives

**FIG. 19.**
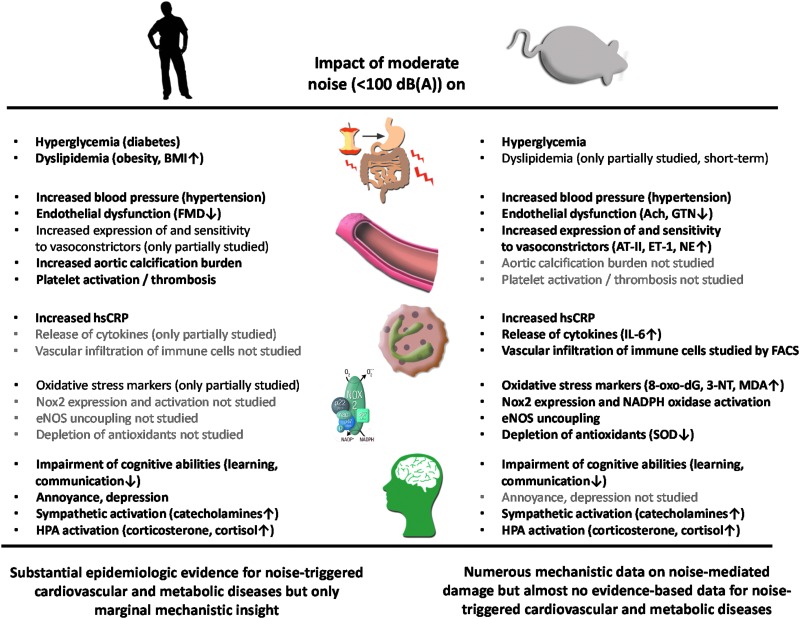
**Overview on human and animal evidence of noise-induced cardiovascular complications.** IL-6, interleukin-6; SOD, superoxide dismutase.

Taken together, the present review summarizes important mechanisms for the development of cardiometabolic diseases in response to exposure to noise (and other environmental stressors). Noise leads to oxidative stress, vascular dysfunction, autonomic imbalance, and metabolic abnormalities, further increasing the adverse health effects of classical risk factors such as arterial hypertension, diabetes, hypercholesterolemia, and smoking (*e.g.*, accelerated progression of atherosclerosis and higher susceptibility to cardiovascular events). While there is substantial clinical symptomatic evidence but only minor mechanistic insight from human studies, there is appreciable experimental animal data available on underlying mechanisms but only few reports on the impact of chronic noise on disease progression in animal models (Fig. 19).

Importantly, both noise and air pollution seem to cause vascular dysfunction by inducing oxidative stress involving partly similar enzymatic pathways, which warrants further preclinical studies addressing this topic. A note of caution with respect to the choice of suitable oxidative stress markers and their interpretation in the context of environmental stressors was published in this Forum (91).

An effective reaction toward reducing environmental transportation noise exposures would require a paradigm shift in human activity and long-term costly mitigation strategies. Given the colocalization of noise and air pollution in big cities and the expected additive benefits of reducing both stressors, an alteration of the current approach, which considers the two exposures separately, may be required. Furthermore, even attempts that address several environmental risk factors simultaneously, changing urban models completely, need to be considered and may lead to cobenefits such as greenhouse gas reduction and increased physical activity of the population. Given the increasing evidence for synergistic/additive effects of air and noise pollution as significant risk factors, their contribution to the pathogenesis of CVD needs to be considered. Accordingly, these environmental stressors should be acknowledged in current guidelines for cardiovascular prevention, acute coronary syndrome, and congestive heart failure.

## Abbreviations Used

BH4: tetrahydrobiopterin
BP: blood pressure
CDA: continuous descent arrivals
cGK-I: cGMP-dependent protein kinase I
cGMP: cyclic guanosine monophosphate
CHF: Swiss Francs
CI: confidence interval
CVD: cardiovascular disease
DALYs: disability-adjusted life years
dB(A): A-weight decibels
EMR: electromagnetic radiation
ENNAH: European Network on Noise and Health
eNOS: endothelial nitric oxide synthase;
ET-1: endothelin-1
EU: European Union
FMD: flow-mediated dilation
FOXO: Forkhead-box-protein
HYENA: Hypertension and Exposure to Noise near Airports
Ihh: Indian hedgehog
IL-6: interleukin-6
JRC: joint research center
L_eq_: average sound pressure level
MACCE: major adverse cardiovascular and cerebral events
NGS: next-generation sequencing
NIHL: noise-induced hearing loss;
^•^NO: nitric oxide
Nox: isoform (*e.g.*, 1, 2, 4) of NADPH oxidase
ONOO^−^: peroxynitrite
PM: particulate matter
RANCH: Road traffic and Aircraft Noise exposure and children's Cognition and Health
ROS: reactive oxygen species
SES: socioeconomic status
sGC: soluble guanylyl cyclase
SOD: superoxide dismutase
SOD2: manganese superoxide dismutase
SPL: sound pressure level
VEGF: vascular endothelial growth factor
VSMCs: vascular smooth muscle cells
WHO: World Health Organization
Ypel2: Yippee-like-2
